# Supplementary material for: Shark discards in selective and mixed-species pelagic longline fisheries
Source: PLoS One. 2020 Aug 31;15(8):e0238595. doi: 10.1371/journal.pone.0238595 (PMC7458300; doi:10.1371/journal.pone.0238595)
Supplement: S1 Table — (DOCX) [file pone.0238595.s001.docx]

**S1 Table.** The numbers of blue and shortfin mako sharks landed per subfleet in 2015 compared to the estimates of retained sharks, those discarded in good and poor condition, respectively including the standard error (S.E.).

|  | **Blue shark** | | | | | | | **Shortfin Mako** | | | | | | |
| --- | --- | --- | --- | --- | --- | --- | --- | --- | --- | --- | --- | --- | --- | --- |
|  | **Landings** | **Retained** | | **Discarded good** | | **Discarded poor** | | **Landings** | **Retained** | | **Discarded good** | | **Discarded poor** | |
|  | **2015. tot** | **Est. tot.** | ***S.E.*** | **Est. tot.** | ***S.E.*** | **Est. tot.** | ***S.E.*** | **2015. tot** | **Est. tot.** | ***S.E.*** | **Est. tot.** | ***S.E.*** | **Est. tot.** | ***S.E.*** |
| Subfleet 1 | 1106 | 1473 | *8* | 22 269 | *116* | 12 043 | *80* | 2201 | 1839 | *9* | 188 | *2* | 180 | *1* |
| Subfleet 2 | 19 348 | 10 495 | *33* | 309 | *3* | 58 882 | *661* | 2731 | 953 | *9* | 62 | *1* | 0 | *0* |
| Subfleet 3 | 34 134 | 33 373 | *223* | 1003 | *7* | 2862 | *19* | 17 590 | 8213 | *51* | 0 | *0* | 334 | *2* |
| Subfleet 4 | 7647 | 8063 | *66* | 97 | *1* | 194 | *2* | 15 424 | 37 335 | *307* | 0 | *0* | 1036 | *9* |
