# Supplementary figures and images for: Shark discards in selective and mixed-species pelagic longline fisheries
Source: PLoS One. 2020 Aug 31;15(8):e0238595. doi: 10.1371/journal.pone.0238595 (PMC7458300; doi:10.1371/journal.pone.0238595)

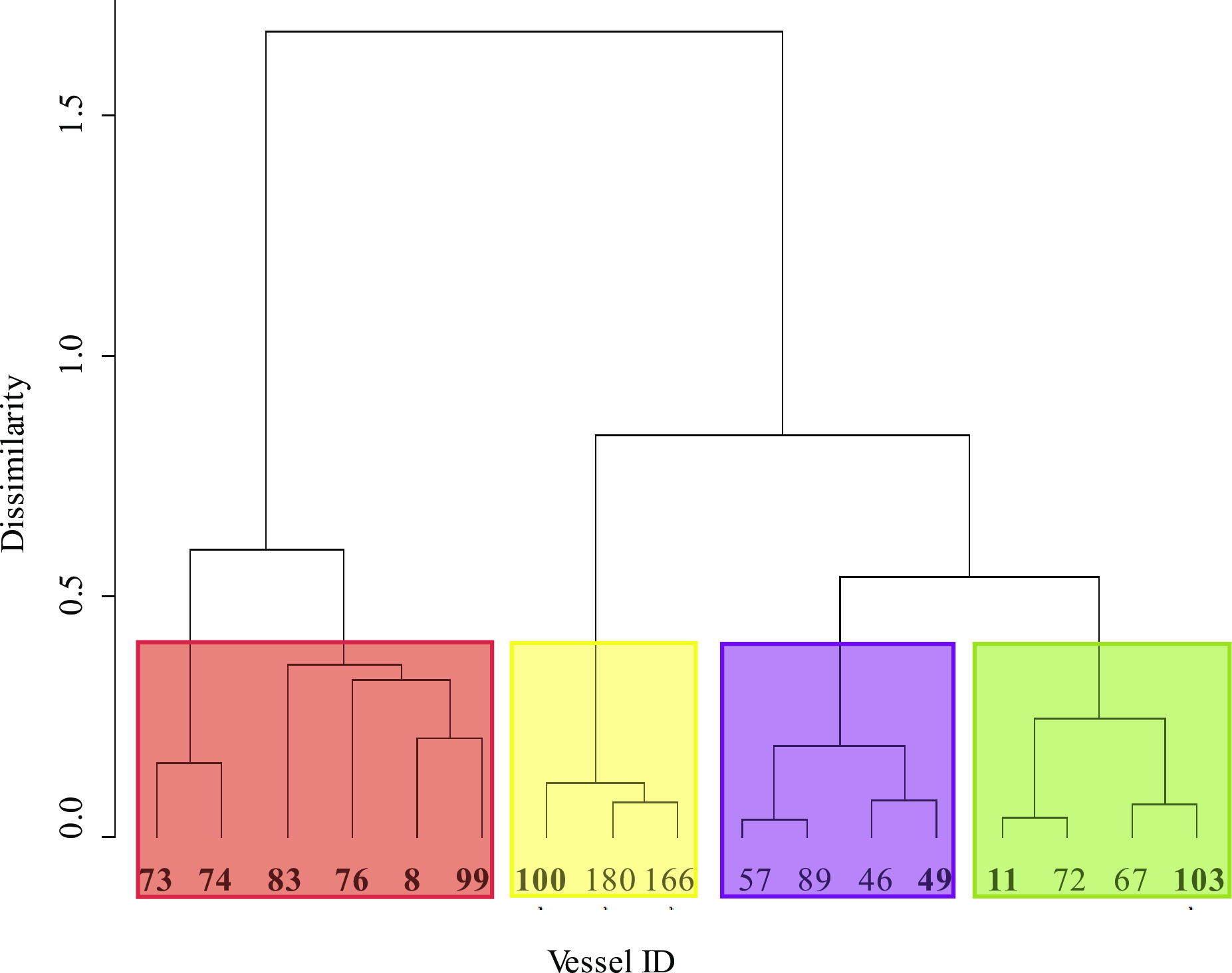

Supplement: S1 Fig — Vessels shown in bold were sampled by a fisheries-independent observer. Subfleet 1 (red) landed mainly tunas and swordfish; Subfleet 2 (green) landed tunas, swordfish and sharks; Subfleet 3 (purple) landed shortfin mako and blue sharks; Subfleet 4 (yellow) landed mainly shortfin makos. (TIF) [file pone.0238595.s002.tif]

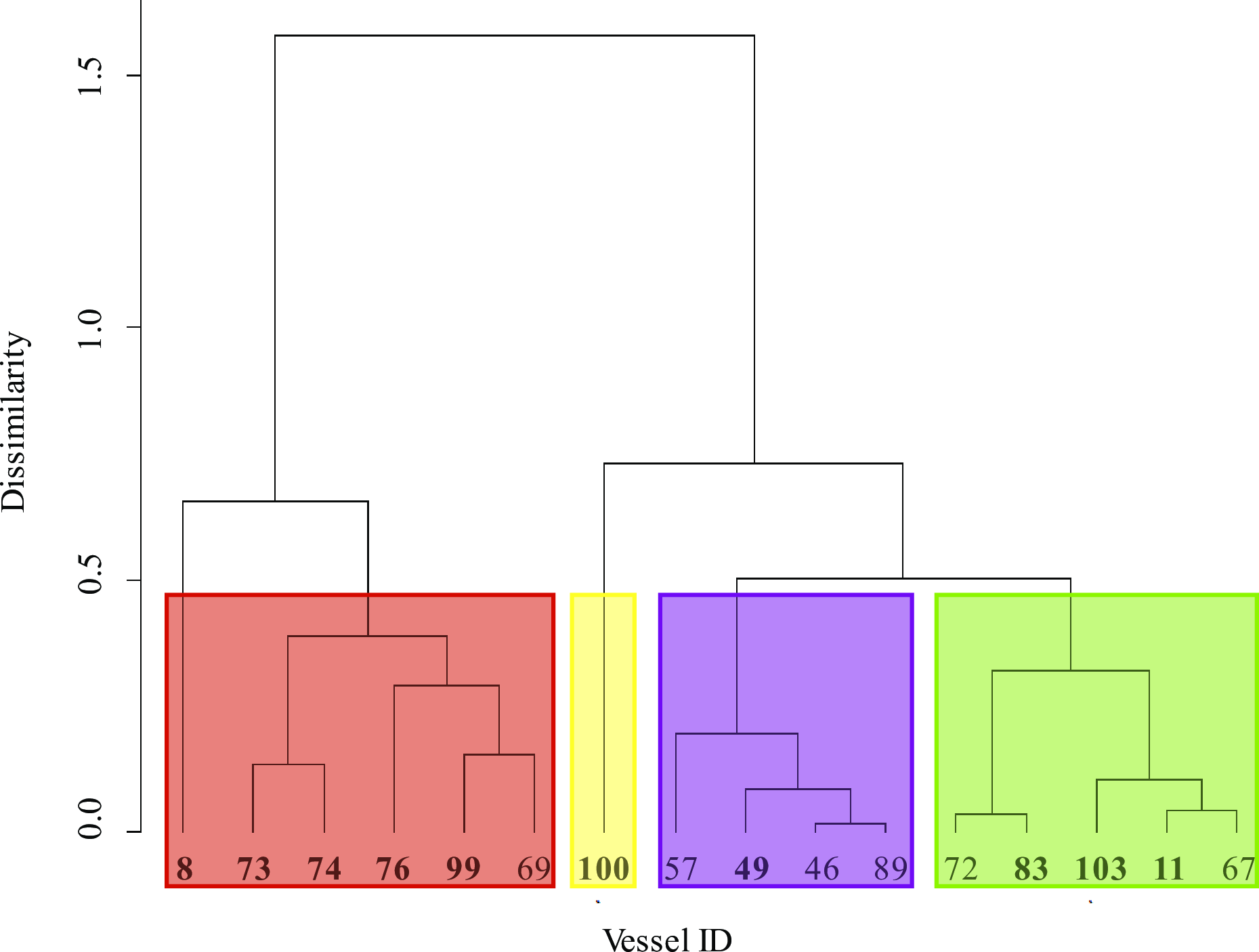

Supplement: S2 Fig — Vessels shown in bold were sampled by a fisheries-independent observer in 2015. Subfleet 1 (red) landed mainly tunas and swordfish; Subfleet 2 (green) landed tunas, swordfish and sharks; Subfleet 3 (purple) landed shortfin mako and blue sharks; Subfleet 4 (yellow) landed mainly shortfin makos. (TIF) [file pone.0238595.s003.tif]

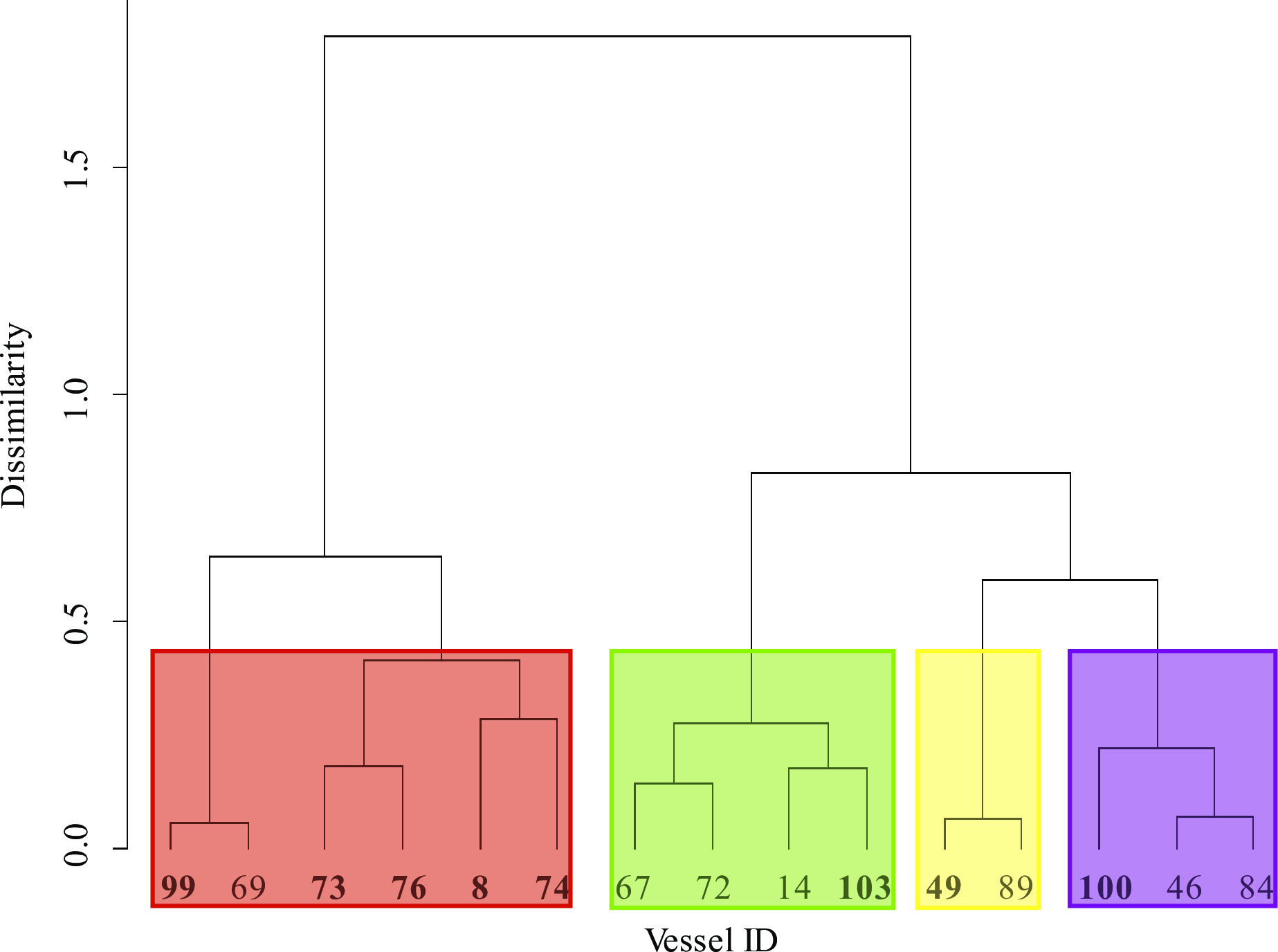

Supplement: S3 Fig — Vessels shown in bold were sampled by a fisheries-independent observer in 2015. Subfleet 1 (red) landed mainly tunas and swordfish; Subfleet 2 (green) landed tunas, swordfish and sharks; Subfleet 3 (purple) landed shortfin mako and blue sharks; Subfleet 4 (yellow) landed mainly shortfin makos. (TIF) [file pone.0238595.s004.tif]
