# Supplementary material for: Shark discards in selective and mixed-species pelagic longline fisheries
Source: PLoS One. 2020 Aug 31;15(8):e0238595. doi: 10.1371/journal.pone.0238595 (PMC7458300; doi:10.1371/journal.pone.0238595)
Supplement: Attachment — Submitted filename: PONE-D-20-12467_reviewer_comments.pdf [file pone.0238595.s007.pdf]

PLOS ONE

Shark discards in selective and mixed-species pelagic longline fisheries

--Manuscript Draft--

**Manuscript Number:**	PONE-D-20-12467
**Article Type:**	Research Article
**Full Title:**	Shark discards in selective and mixed-species pelagic longline fisheries
**Short Title:**	Shark discards in pelagic longline fisheries
**Corresponding Author:**	Gareth Lee Jordaan, MSc<br>South African Association for Marine Biological Research<br>Durban, KwaZulu-Natal SOUTH AFRICA
**Keywords:**	Blue shark; Shortfin mako shark; Pelagic longline fishery; Fishery discards; Management regulations; Fishing mortality
**Abstract:**	The conservation status of sharks is considered vulnerable with declining populations for several species, yet data on shark fishing mortality remain limited. Pelagic sharks are increasingly retained by mixed-species fisheries, or are discarded and not reported by selective fisheries for tunas or swordfish. We estimated the fishing mortality of sharks (landings plus discard mortalities) in a South African-flagged pelagic longline fishery with diverse targeting and discard behaviour. A hierarchical cluster analysis was used to stratify the fleet according to fishing behaviour, based on the relative proportions of tunas, swordfish, blue sharks and shortfin makos landed by individual vessels between 2013 and 2015. A spatial analysis of logbook data indicated that subfleets operated in distinct fishing areas, with overlap. Fisheries observers sampled ~5% of commercial longlines set during 2015, and recorded the species, discard ratios and physical condition of 6 019 captured sharks. Blue sharks and shortfin makos dominated observed shark catches, which comprised of nine species and two species groups. Some 47% of observed sharks were retained and 20% were discarded in good physical condition. Only 4% of shortfin makos were discarded, compared to 68% of blue sharks. Blue shark discard mortality rates were twice as high as published at-vessel mortality rates, suggesting onboard handling as a major cause of mortality. Extrapolation to total fishing effort indicated a near 10-fold increase in blue shark and shortfin mako fishing mortality compared to an earlier study (1998-2005). Escalating shortfin mako fishing mortality was attributed to increased targeting to supply higher market demand. Discarding of blue sharks by selective fishing for tunas and swordfish had a greater impact on their fishing mortality than retention by shark-directed subfleets. Higher levels of observer sampling are required to increase confidence in discard ratio estimates.
**Order of Authors:**	Gareth L. Jordaan, MSc
	Jorge Santos
	Johan C. Groeneveld
**Additional Information:**	
**Question**	**Response**
**Financial Disclosure**<br><br>Enter a financial disclosure statement that describes the sources of funding for the work included in this submission. Review the submission guidelines for detailed requirements. View published research articles from *PLOS ONE* for specific examples.<br><br>This statement is required for submission	This work was funded by National Research Foundation (NRF) incentive fund (grant number 96309) to Johan Groeneveld, as well as through a college bursary provided by the University of KwaZulu-Natal (UKZN). The funders had no role in study design, data collection and analysis, decision to publish, or preparation of the manuscript.

Powered by Editorial Manager® and ProduXion Manager® from Aries Systems Corporation

and **will appear in the published article** if the submission is accepted. Please make sure it is accurate.<br><br>**Unfunded studies**<br>Enter: *The author(s) received no specific funding for this work.*<br><br>**Funded studies**<br>Enter a statement with the following details:<br>• Initials of the authors who received each award<br>• Grant numbers awarded to each author<br>• The full name of each funder<br>• URL of each funder website<br>• Did the sponsors or funders play any role in the study design, data collection and analysis, decision to publish, or preparation of the manuscript?<br>• **NO** - Include this sentence at the end of your statement: *The funders had no role in study design, data collection and analysis, decision to publish, or preparation of the manuscript.*<br>• **YES** - Specify the role(s) played.<br><br>* typeset	
**Competing Interests**<br><br>Use the instructions below to enter a competing interest statement for this submission. On behalf of all authors, disclose any competing interests that could be perceived to bias this work—acknowledging all financial support and any other relevant financial or non-financial competing interests.<br><br>This statement **will appear in the published article** if the submission is accepted. Please make sure it is accurate. View published research articles from *PLOS ONE* for specific examples.	The authors have declared that no competing interests exist.

**NO authors have competing interests**<br><br>Enter: *The authors have declared that no competing interests exist.*<br><br>**Authors with competing interests**<br><br>Enter competing interest details beginning with this statement:<br><br>*I have read the journal's policy and the authors of this manuscript have the following competing interests: [insert competing interests here]*<br><br>* typeset	
**Ethics Statement**<br><br>Enter an ethics statement for this submission. This statement is required if the study involved:<br><br>• Human participants<br>• Human specimens or tissue<br>• Vertebrate animals or cephalopods<br>• Vertebrate embryos or tissues<br>• Field research<br><br>Write "N/A" if the submission does not require an ethics statement.<br><br>General guidance is provided below. Consult the submission guidelines for detailed instructions. **Make sure that all information entered here is included in the Methods section of the manuscript.**	N/A

Powered by Editorial Manager® and ProduXion Manager® from Aries Systems Corporation

**Format for specific study types**<br><br>**Human Subject Research (involving human participants and/or tissue)**<br>• Give the name of the institutional review board or ethics committee that approved the study<br>• Include the approval number and/or a statement indicating approval of this research<br>• Indicate the form of consent obtained (written/oral) or the reason that consent was not obtained (e.g. the data were analyzed anonymously)<br><br>**Animal Research (involving vertebrate animals, embryos or tissues)**<br>• Provide the name of the Institutional Animal Care and Use Committee (IACUC) or other relevant ethics board that reviewed the study protocol, and indicate whether they approved this research or granted a formal waiver of ethical approval<br>• Include an approval number if one was obtained<br>• If the study involved *non-human primates*, add *additional details* about animal welfare and steps taken to ameliorate suffering<br>• If anesthesia, euthanasia, or any kind of animal sacrifice is part of the study, include briefly which substances and/or methods were applied<br><br>**Field Research**<br><br>Include the following details if this study involves the collection of plant, animal, or other materials from a natural setting:<br>• Field permit number<br>• Name of the institution or relevant body that granted permission	
**Data Availability**<br><br>Authors are required to make all data underlying the findings described fully available, without restriction, and from the time of publication. PLOS allows rare exceptions to address legal and ethical concerns. See the PLOS Data Policy and FAQ for detailed information.	Yes - all data are fully available without restriction

Powered by Editorial Manager® and ProduXion Manager® from Aries Systems Corporation

A Data Availability Statement describing where the data can be found is required at submission. Your answers to this question constitute the Data Availability Statement and **will be published in the article**, if accepted.<br><br>**Important:** Stating 'data available on request from the author' is not sufficient. If your data are only available upon request, select 'No' for the first question and explain your exceptional situation in the text box.<br><br>Do the authors confirm that all data underlying the findings described in their manuscript are fully available without restriction?	
**Describe where the data may be found in full sentences. If you are copying our sample text, replace any instances of XXX with the appropriate details.**<br><br>• If the data are **held or will be held in a public repository**, include URLs, accession numbers or DOIs. If this information will only be available after acceptance, indicate this by ticking the box below. For example: *All XXX files are available from the XXX database (accession number(s) XXX, XXX.).*<br>• If the data are all contained **within the manuscript and/or Supporting Information files**, enter the following: *All relevant data are within the manuscript and its Supporting Information files.*<br>• If neither of these applies but you are able to provide **details of access elsewhere**, with or without limitations, please do so. For example:<br><br>*Data cannot be shared publicly because of [XXX]. Data are available from the XXX Institutional Data Access / Ethics Committee (contact via XXX) for researchers who meet the criteria for access to confidential data.*<br><br>*The data underlying the results presented in the study are available from (include the name of the third party*	All relevant data are within the manuscript and its Supporting Information files.

and contact information or URL).<br>• This text is appropriate if the data are owned by a third party and authors do not have permission to share the data.<br><br>* typeset	
Additional data availability information:	

Manuscript

Click here to download Manuscript Jordaan_et_al._Manuscript.docx

Shark discards in selective and mixed-species pelagic longline fisheries

Gareth L. Jordaan[1*¶], Jorge Santos[2¶] and Johan C. Groeneveld[1,3¶]

[1]Oceanographic Research Institute, 1 King Shaka Avenue, Durban, South Africa

[2]Norwegian College of Fishery Science, University of Tromsø, NO-9037, Tromsø, Norway

[3]School of Life Sciences, University of KwaZulu-Natal, Pietermaritzburg, KwaZulu-Natal, South Africa

*Corresponding author:

E-mail: jordaan.gareth@gmail.com

[¶]These authors contributed equally to this work.

Abstract

The conservation status of sharks is considered vulnerable with declining populations for several species, yet data on shark fishing mortality remain limited. Pelagic sharks are increasingly retained by mixed-species fisheries, or are discarded and not reported by selective fisheries for tunas or swordfish. We estimated the fishing mortality of sharks (landings plus discard mortalities) in a South African-flagged pelagic longline fishery with diverse targeting and discard behaviour. A hierarchical cluster analysis was used to stratify the fleet according to fishing behaviour, based on the relative proportions of tunas, swordfish, blue sharks and shortfin makos landed by individual vessels between 2013 and 2015. A spatial analysis of logbook data indicated that subfleets operated in distinct fishing areas, with overlap. Fisheries observers sampled ~5% of commercial longlines set during 2015, and recorded the species, discard ratios and physical condition of 6 019 captured sharks. Blue sharks and shortfin makos dominated observed shark catches, which comprised of nine species and two species groups. Some 47% of observed sharks were retained and 20% were discarded in good physical condition. Only 4% of shortfin makos were discarded, compared to 68% of blue sharks. Blue shark discard mortality rates were twice as high as published at-vessel mortality rates, suggesting onboard handling as a major cause of mortality. Extrapolation to total fishing effort indicated a near 10-fold increase in blue shark and shortfin mako fishing mortality compared to an earlier study (1998-2005). Escalating shortfin mako fishing mortality was attributed to increased targeting to supply higher market demand. Discarding of blue sharks by selective fishing for tunas and swordfish had a greater impact on their fishing mortality than retention by shark-directed subfleets. Higher levels of observer sampling are required to increase confidence in discard ratio estimates.

Introduction

Pelagic longline fisheries for tunas and swordfish typically have high incidental catches of sharks, of which most are discarded overboard as unwanted catch [1, 2]. Hooked sharks often die during capture or shortly thereafter as a result of physical injuries or physiological stress, and high post-discard mortality rates have been found for sharks released alive [3-6]. Discarded sharks are rarely reported in fisher logbooks [1], and their numbers, species composition and associated fishing mortality are therefore poorly known.

Not all captured sharks are discarded. Several species are increasingly retained or have become secondary target species of pelagic longline fisheries [7]. Shark meat markets have shown an upwards trend over the past decades [8], and the demand for shark fins remains high [9]. Landings of some shark species have increased as a result of targeting [10], even though global shark and ray landings have declined by 20% since 2003, mainly as a result of fishing pressure [11]. Landings data alone grossly under-represent shark fishing mortality associated with pelagic longline fisheries, because it discounts discard mortalities. More accurate estimates of fishing mortality rates and levels, important for stock status assessments, can only be obtained by combining landings and discard mortality estimates [1, 2].

Advances in gear technology have increased the selectivity of longline fishing gear, which can easily be adapted to target specific species groups [12]. Vessels can switch between fishery targets by setting hooks at different depths or times [5, 13], changing fishing locations [10], replacing gear components such as leaders on hooks [14] or by using different bait types [15, 16]. He et al. [17] segregated dissimilar types of fishing effort in a Hawaiian-based pelagic longline fishery, based on a cluster analysis of the species composition of landings. The approach distinguished between selective fishing for tunas or swordfish, and mixed-species fishing that included pelagic sharks. Clear differences in fishing strategies between vessel

clusters or subfleets were revealed when the composition of landings was matched with the spatial distribution of longline sets.

Targeting of sharks and discard practices vary substantially across fishing fleets, in response to market demand or regulatory measures [2]. James et al. [18] showed that shark species (as a proxy for economic value) is a key determinant of whether a shark is retained and processed or discarded, and that country (a proxy for regulatory environment) was also important. Operationally, factors that affect the decision to retain or discard sharks are the abundance of other target species, whether sharks are damaged or too small to process, availability of freezer space, retention or trade bans, or output controls such as upper catch limits [2, 6, 18]. Mandatory release regulations operate in some longline fisheries [19], but in others there are landing obligations for species shown to have high potential for discard mortality [4].

Pelagic shark populations are typically vulnerable to overfishing because of life history traits that include slow growth, few offspring which mature late, and a long natural lifespan [13, 20, 21]. They migrate freely and widely over their range [22, 23] making them vulnerable to both high seas fishing fleets and local fleets that operate closer to the coast. The conservation status of several species is considered to be vulnerable with decreasing population trends (www.iucnredlist.org) and some species are listed on CITES Appendix II (www.cites.org) which limits international trade.

In spite of the vulnerability of pelagic sharks to longline fishing, the quality and availability of reliable data on shark fishing mortality remain extremely limited [2], compromising efforts to determine stock status. Campana et al. [1] highlighted the importance of independent observer programmes to collect quantitative information on catch- and discard rates by species, gear type and ocean region, to allow for more accurate estimates of fishing mortality for pelagic sharks.

The South African-flagged pelagic longline fishery lands tunas, swordfish and pelagic sharks caught in the coastal Southeast (SE) Atlantic and Southwest (SW) Indian Ocean. The bulk of blue sharks *Prionace glauca* and shortfin makos *Isurus oxyrinchus* landed by the fishery originate from the temperate south and west coasts of South Africa, with lesser quantities captured along the subtropical east coast [10, 24]. Sharks have been managed as a bycatch of the fishery since 2005 [19] and landings are reported to the International Commission for the Conservation of Atlantic Tunas (ICCAT; www.iccat.int) and the Indian Ocean Tuna Commission (IOTC; www.iotc.org) respectively. Shortfin mako landings and catch per unit effort (CPUE) have increased in recent years, suggesting increased targeting and/or retention as part of a mixed-species fishing strategy [10]. Blue shark CPUE has varied, suggesting occasional targeting and retention, interspersed with periods when blue sharks were not caught or discarded and not reported.

A recent review of formal management protocols for sharks specified in a National Plan of Action (NPOA; [25]) recognized that management so far has been on an *ad hoc* basis [26]. The estimation of shark discards was highlighted as a weakness, because there were insufficient data to enable quantification of shark mortalities associated with bycatch. We addressed this limitation for blue sharks and shortfin makos captured by the South African-flagged pelagic longline fishery by estimating shark fishing mortality, as the sum of reported landings and discard mortalities estimated from data collected at sea by independent fisheries observers.

Materials and methods

Study area

The South African exclusive economic zone (EEZ; Fig 1) was stratified into four geographical areas: West (cool temperate waters influenced by the Benguela Current; Namibian border to 33°S); Southwest (dynamic boundary zone between the Benguela Current and subtropical Agulhas Current systems, including the western Agulhas Bank; 33°S – 20°E); South (lower Agulhas Current area, where the narrow shelf broadens towards the west to form the eastern Agulhas Bank; 20 – 26°E); and East (subtropical waters influenced by the upper Agulhas Current; 26°E to Mozambique border). The four areas covered the main commercial fishing grounds used by the South African-flagged pelagic longline fleet (Fig 1) and conformed to a spatial framework used by Petersen et al. [24] and Jordaan et al. [10] to study shark bycatches of the fishery.

Fig 1. Spatial distribution of sampled longlines per trip (coloured dots representing individual trips; n = 89 longlines) and of all longlines set in 2013-2015 (grey dots). Fishing areas were West and Southwest (SE Atlantic), South and East (SW Indian Ocean) [10, 24]. The boundary at 20˚E separated fishing areas reporting to the IOTC and ICCAT. The South African Exclusive Economic Zone (EEZ) extends 200 nm from the shore.

Landings- and logbook data

Landings data on a per-trip basis were obtained from the Department of Environment, Forestry and Fisheries (DEFF) for South African-flagged pelagic longline vessels for 2013–2015. The data comprised of the numbers and total weight of retained fish and sharks, categorized to

species level, or to species groups for less common or similar-looking species. Blue sharks and shortfin makos dominated shark landings. Sharks that could not be identified to species level were grouped as requiem sharks (mostly *Carcharhinus* spp.), threshers (*Alopias* spp.), hammerheads (*Sphyrna* spp.) or as unidentified sharks.

We checked the consistency of landings data by dividing the total weight of landings per vessel by the numbers of fish or sharks reported, to obtain average individual weights. Average weights remained within plausible bounds for tunas (17–39 kg per fish), swordfish (40–100 kg) Shortfin makos (6–37 kg) and blue sharks (5–28 kg). Further, we regressed the recorded numbers per species group against their respective discharge weights for individual vessels expecting that landed weight would increase concurrently with increasing numbers. Least squares linear regressions confirmed strong positive relationships (n = 17 vessels; $r^2 > 0.9$; $p < 0.001$) for all four species groups tested. The tests confirmed that the landings data were of a consistent quality.

To obtain information on the spatial and temporal distribution of fishing effort, logbook records of individual fishing vessels (numbers of hooks set per day, set and haul positions) were extracted from the DEFF database. Retained catches per species group were also recorded in logbooks, but discarded fish and sharks were not recorded. The dataset was cleaned by removing anomalous records in which fishing effort or catch composition were clearly incorrect or mismatched, as described in detail by Jordaan et al. [10].

Fleet stratification using cluster analysis

We used a hierarchical cluster analysis and dendrograms in the statistical package R, version 3.3.2 [27] to stratify the pelagic longline fleet into subfleets, based on the relative proportions of tunas, swordfish, blue sharks and shortfin makos landed between 2013 and 2015. The

'hclust' function of the 'fastcluster' package in R [28] was used, relying on Euclidian distances between categories for each vessel and complete linkages [29]. We experimented with three and four *a priori* clusters in the dendrograms, and also explored hierarchical clustering among subsets of vessels landing mainly sharks.

A K-medoids clustering approach ('pam' function from the R-package 'cluster' [30]) was conducted independently from the hierarchical analysis and produced similar clusters (https://www.datanovia.com/en/lessons/k-medoids-in-r-algorithm-and-practical-examples/) [31] for the combined 2013 to 2015 data. An optimal fit of four clusters was derived from the average silhouette method ('fviz_cluster' function in R-package 'factoextra' [32]).

Individual cluster plots were generated for landings data in each year separately, to dynamically regroup vessels based on their annual landings. Movements of individual vessels between subfleets in successive years (i.e. change of fishing behaviour based on landings information) could therefore be traced.

Observer sampling

Fisheries observers sampled the numbers of hooks hauled, and numbers of tunas, swordfish, and sharks caught during commercial operations. Observer placement depended on opportunity and space availability on fishing vessels and was not randomly distributed. Nevertheless, different fishing vessels were sampled to facilitate broad coverage of discard practices and to ensure that all geographical areas and seasons were represented (Table 1). Sharks were identified to species level based on standard species identification guides [33]. Sharks were categorized as retained (kept and processed) or discarded (thrown overboard or branchlines cut adjacent to the vessel). Discarded sharks were further categorized as dead, alive in poor physical condition (debilitating injuries resulting from hooking in the eye or gills, clubbing,

cutting or gaffing during handling, or motionless and unable to swim) or alive in good physical condition (shark clearly active, with no or minor physical injuries, such as a hook lodged in the jaw or mouth).

Table 1. Observer sampling effort per subfleet as number of sampling trips, quarter year covered, longlines and hooks sampled, and observed catches as numbers of tunas and swordfish, shortfin makos, blue sharks and other sharks.

	Sampling Effort					Observed Catch (numbers)			
**Fleet**	**Trips (n)**	**Quarters covered**	**Longlines (n)**	**Hooks (n)**	**% hooks sampled**	**Tuna, Swordfish**	**Shortfin mako**	**Blue shark**	**Other sharks**
Subfleet 1	7	1 - 4	63	45 925	56	1 048	101	1 743	101
Subfleet 2	2	2, 4	8	6 524	61	13	19	1 281	3
Subfleet 3	1	1	9	10 200	80	21	230	1 002	14
Subfleet 4	1	1	9	8 454	71	0	1 185	258	124
**All vessels**	**11**		**89**	**71 103**	**61**	**1 082**	**1 535**	**4 284**	**242**

Quarter years are January to March (1), April to June (2), July to September (3) and October to December (4).

The observer data were used to calculate a catch ratio (number of sharks / number of sampled hooks) per vessel for retained sharks and for those in the three discard fate categories (i.e. discard ratios for sharks that were dead, alive in poor condition, or alive in good condition) respectively. To obtain catch ratios for unsampled vessels, data from sampled vessels were averaged, as follows:

$$\bar{y_t} = \frac{\sum^{n} y_i}{n} \quad (1) \qquad s_i = \sqrt{\frac{\sum_{i=1}^{n} (y_i - \bar{y})^2}{n-1}} \quad (2)$$

Where y_i is the discard ratio for vessel i, $\bar{y_t}$ is the average ratio and s_i the standard deviation of catch per hook for each shark species and fate combination per subfleet. Catch ratios were only determined for 2015, because observer data were restricted to that year. Catch ratios were assumed to remain constant for extrapolations based on 2013 and 2014 fishing effort.

Catch ratios were used to scale observed numbers of fish and sharks up to the total fishing effort based on logbooks, accounting for subfleet and discard fate category, as follows:

$$\hat{t}_{ST} = \sum_{i=1}^{L} N_i \bar{y}_t \qquad (3) \qquad \hat{V}(\hat{t}_{ST}) = \sum_{i=1}^{L} N^2{}_i \left(\frac{N_i - n_i}{N_i}\right) \frac{s^2{}_i}{n_i} \qquad (4)$$

where $\hat{t}_{ST}$ is the stratified total numbers with variance, $\hat{V}(\hat{t}_{ST})$ for each species and discard fate category [34], *Ni* is the total number of hooks hauled per year, and *ni* are the numbers of hooks observed at sea. In the equation, fishing effort (*Ni*) could be reassigned between subfleets in each year between 2013 and 2015, to recreate the effects of vessel movements between subfleets. Reassignment took place on a vessel basis (number of hooks set by individual vessel moved to appropriate subfleet categories in each year).

The accuracy of the estimation method was determined as the ratio of retained sharks (*R*, estimates obtained by raising observer counts to total fishing effort) to landed sharks (*L*, numbers of sharks landed, obtained from DEFF landings data). The ratio *R/L* = 1.0 then indicates that retained sharks equal reported landings; R/L >1.0 indicates an overestimate, and R/L < 1.0 an underestimate.

Results

Cluster analysis and spatio-temporal distribution of fishing effort

The cluster analysis of landings data recovered four subfleets based on the combined 2013 to 2015 data (n = 20 vessels): vessels that landed tunas and swordfish, but few sharks (subfleet 1; n = 6 vessels); vessels that landed tunas, swordfish and sharks (subfleet 2; n = 6); vessels that landed blue sharks and shortfin makos (subfleet 3; n = 4); and vessels that landed mainly

shortfin makos (subfleet 4; n = 4) (Fig 2). Clustering of landings for each year individually recovered the same four subfleets in all cases, with some vessels in each year exiting or entering the fishery, or moving between subfleet categories (Supplementary data, S1-S3 Figs). The numbers of active vessels per year were 16 in 2013 and 2014, and 17 in 2015. The cluster analysis therefore supported a distinct fishing behaviour per subfleet, based on the relative composition of landings.

Fig 2. Dendrogram of the vessels (n = 20) clustered into four subfleets based on the species composition of landings (2013-2015). Vessels in bold were sampled by the observer. Subfleet 1 (red) landed mainly tunas and swordfish; Subfleet 2 (green) landed tunas, swordfish and sharks; Subfleet 3 (purple) landed shortfin mako and blue sharks; Subfleet 4 (yellow) landed mainly shortfin makos. Vessel 83 fished in subfleet 1 in 2015, when it was sampled by the observer (see S1 Fig).

A spatial analysis of fishing effort (numbers of hooks set) recorded in logbooks (n = 4.97 million hooks for 2013-2015 pooled data) showed that subfleets operated in distinct fishing areas. Subfleet 1 (tunas and swordfish) set >50% of hooks in the East, but no other subfleet fished in that area (Table 2). Subfleet 4 (makos) set most hooks in the South (58%) and Southwest (38%), whereas subfleet 3 (blue sharks and makos) set 77% of its hooks in the Southwest. Vessels that fished in the West (27% of hooks set by subfleet 1 and 52% set by subfleet 2) landed mainly tunas and swordfish, with a lesser proportion of sharks. Overall, tuna- and swordfish directed vessels (subfleets 1 and 2) fished mainly in the East and West fishing areas, whereas shark-directed vessels (subfleets 3 and 4) fished predominantly in the South and

Southwest. There remains considerable overlap between the spatial fishing pattern and landings, which could not be resolved at the coarse spatial scale used in the analysis.

Table 2. Spatial distribution of fishing effort per subfleet as percentage of hooks set per fishing area, based on logbook data from 2013-2015 (n = 4.97 million hooks).

Fleet	Composition of landings	West	Southwest	South	East
Subfleet 1	Tuna & swordfish	26-50	10-25	<10	51-75
Subfleet 2	Tunas, swordfish & sharks	51-75	26-50	<10	<10
Subfleet 3	Makos & blue sharks	10-25	76-100	<10	<10
Subfleet 4	Makos	<10	26-50	51-75	<10

Seasonal distribution of fishing effort differed among the four subfleets (Fig 3). Subfleets 1 and 2 set fewest hooks in Jan-Mar (16% and 8% of annual effort, respectively) with the bulk of their fishing effort concentrated in Apr-Sep (59% and 68% of hooks set) to target tunas during winter months. Fishing effort of subfleet 3 (blue sharks and makos) was sharply down in Jul-Sep (13% of annual effort) but remained relatively consistent between 26% and 33% per quarter during the rest of the year. Fishing effort of subfleet 4 (makos) was consistent throughout the year, remaining within a narrow band of 23 – 28% of hooks set per quarter year.

Fig 3. Seasonal distribution of fishing effort by individual subfleets as the proportional numbers of hooks set per quarter year, based on 2013 - 2015 logbook data (n = 4.97 million hooks).

Observer samples

Observer sampling took place in 2015, with an additional sampling trip (4 lines) undertaken in 2018. Observers sampled 71 102 of 116 872 hooks (61%) set along 89 longlines during 11 trips at sea (Table 1), on 10 different fishing vessels. All four subfleets were sampled although sampling intensity was unequal. The bulk of sampling was directed at subfleet 1 (65% of sampled hooks), where seven sampling trips were undertaken on six different vessels (one vessel was sampled twice). Two of four active vessels in subfleet 2 were sampled (9% of hooks); one of four in subfleet 3 (14% of hooks), and one of three in subfleet 4 (12% of hooks). By area, most hooks were sampled in the East (45% of hooks sampled), followed by 29% in the South, and 13% in the Southwest and West, respectively. Observer coverage in the East was limited to the northern half of that fishing area, consistent with the spatial distribution of commercial fishing effort (Fig 1). By quarter year, four sampling trips were undertaken in January-March, two in April-June, one in July-September, three in October-December, and one trip started in June (end of quarter 2 and ended in July (beginning of quarter 3) (Table 1)). Overall, some 5% of all commercial longlines set during 2015 (n = 1 699 lines set) were sampled.

The observed catches in the West and Southwest fishing areas comprised mainly of blue sharks (≥88% by numbers) and smaller quantities of shortfin makos, tunas and swordfish (Fig 4). By contrast, makos dominated catches in the South (58%), but blue sharks remained relatively abundant (36%). In the East, mostly tunas (43%), swordfish (30%) and blue sharks (26%) were observed, with few makos. Blue sharks were common in all four areas, and made up the bulk of the observed catch in the pooled data (62% by numbers) followed by makos (22%). Numerically, tunas and swordfish combined made up only 16% of the observed catches.

Fig 4. Catch composition of key species groups per fishing area based on observer samples. Sample size (number of sharks) per area is shown.

Observers identified 6 019 sharks, comprising of nine species and two species groups (hammerheads and thresher sharks) (Fig 5). Blue sharks (71%) and shortfin makos (25%) dominated shark catches, and of the remainder, bronze whaler *Carcharhinus brachyurus* (2%), silky sharks *C. falciformis* (1%) and thresher sharks *Alopias* spp. (1%) were present in smaller numbers. Other shark species were infrequently caught, making up <1% of all sharks observed.

Fig 5. Proportion of sharks per species or species group that were retained, discarded dead, in poor condition and in good condition, based on observer samples. Sample size per species is shown.

Of all hooked sharks, 47% were retained, 31% were discarded dead, 2% in poor physical condition, and 20% in good condition (Fig 5). Some 96% of shortfin makos and 32% of blue sharks were retained and processed, and the remainder discarded overboard. All other sharks were discarded, except for bronze whaler sharks, of which five individuals (5%) were retained by one vessel. Discard mortality rates differed between species (Fig 5). The bulk of discarded makos were dead (82%), and most discarded silky- and thresher sharks were either dead (67% in both cases) or in poor condition (5%, respectively). Of 2 877 discarded blue sharks, 58% were dead and a further 4% in poor condition. Some 51% of bronze whalers, 54% of porbeagle, 32% of hammerheads and 25% of oceanic whitetip sharks were dead when discarded. Crocodile sharks were more hardy, and although none were dead when discarded, one

individual (<10%) was in a poor condition. A single dusky- and a tiger shark were captured, ~~respectively,~~ and discarded in good condition.

Estimation model

R/L ratios were first compared between estimation models performed on unstratified data (single fishing fleet with homogenous fishing behaviour assumed) and stratified data (subfleets with heterogenous behaviour, based on outcome of cluster analysis) (Table 3). Stratification improved the accuracy of blue shark estimates, by increasing the R/L ratio from 0.46 to 0.86, relative to the benchmark DEFF landings data. For shortfin makos, the R/L ratio increased from 0.75 for the unstratified estimate to 1.27 for the estimate based on a stratified fleet. Stratification therefore increased the importance of makos in estimates. The over-estimation of mako numbers after stratification (R/L = 1.27) suggests that observers sampled trips with atypically high mako catches, especially in subfleets 3 (blue sharks and makos) and 4 (makos). The bias is therefore attributed to under-sampling (a single trip sampled in subfleets 3 and 4, respectively; Table 1). Despite the bias, the relative accuracy of the estimates confirmed that catch ratios derived from observer data could successfully be combined with fishing effort data from commercial logbooks to reconstruct the quantities of sharks discarded without records.

Table 3. Accuracy of reconstructed estimates of retained sharks relative to reported landings for unstratified versus stratified fleets.

Shark species	Landings in 2015 (n)	Unstratified est. (n)	R/L ratio	Stratified est. (n)	R/L ratio
Blue shark	62 235	28 464	0.46	53 404	0.86
Shortfin mako shark	37 946	28 496	0.75	48 340	1.27

A ratio of estimated retained catch to landings (R/L ratio) of 1.0 signifies equality, >1.0 indicates an overestimate, and < 1.0 an underestimate.

Reconstructions of retained sharks by individual subfleets were more variable than for the pooled data. For subfleet 1, estimates were closest to benchmark landings data for blue sharks (R/L = 1.33) and shortfin makos (0.84) (Figs 6a and b), most likely because observer sampling was more extensive (7 trips on 6 vessels) and covered all four seasonal quarters. For this section, sharks discarded in poor physical condition included those sharks that were discarded dead plus those sharks discarded in a poor physical condition, as we assumed that they were not likely to survive. The estimates confirmed that few makos were caught by subfleet 1 (est. 2 206 makos caught), and that only 17% of them were discarded, often dead or in a poor physical condition (est. 368 makos discarded, 49% in poor condition). Conversely, subfleet 1 caught blue sharks in large numbers (est. 35 785) but discarded 96% of them. Of the discarded blue sharks, 35% were dead or in a poor physical condition. Even though subfleet 1 landed mainly tunas and swordfish, discarding of incidentally caught blue sharks, often dead or in a poor physical condition, indicated high fishing mortality of blue sharks.

Fig 6. Numbers of blue and shortfin mako sharks landed (L) and estimates of sharks retained (R), discarded in good condition and discarded in poor condition (assumed dead) for subfleet 1 (a and b), subfleet 2 (c and d), subfleet 3 (e and f) and subfleet 4 (g and h) based on observer samples, and raised to total fishing effort per subfleet. The R/L ratio (estimated retained / reported landed) is provided as a measure of accuracy. A R/L ratio of 1.0 signifies equality; >1.0 is an overestimate and <1.0 is an underestimate.

For subfleet 2, the model underestimated retained catches of blue sharks (R/L = 0.54) and shortfin makos (0.35) relative to landings data (Figs 6c and d). Few makos were caught or discarded, but the numbers of blue sharks caught were exceptionally high (est. 69 687) of which

87% were discarded, nearly all of them dead or in a poor physical condition. Despite landing sharks together with tunas and swordfish, the high incidental catches of blue sharks that were discarded overboard indicated that subfleet 2 also had a high impact on blue sharks.

For subfleet 3, the model accurately estimated retained blue shark catches relative to landings data (R/L = 0.98) but underestimated retained catches of shortfin makos by about half (0.47) (Figs 6e and f). For subfleet 4, the retained blue shark estimate was also accurate (1.05), but makos were grossly overestimated relative to landings (2.42) (Figs 6g and h). Fewer observer samples from which to derive catch ratios, and sampling of too few vessels in subfleets 2 to 4, compared to subfleet 1, could explain the higher variability and reduced accuracy of estimates. Subfleets 3 and 4 landed mainly sharks, and consequently few captured makos (4% and 3%, respectively) or blue sharks (12% and 3%) were discarded. The greater proportion of makos than blue sharks landed by subfleet 4, compared to subfleet 3, was because more makos were caught by subfleet 4, rather than an increase in blue shark discards. A spatial effect is therefore implied, in which subfleet 4 operated in fishing areas where makos were more abundant than blue sharks, i.e. in the South area.

Estimates of the numbers of shortfin makos discarded by the fleet remained stable at low levels between 2013 and 2015, but blue shark discards were high in all three years and increased moderately to a peak of approximately 100 000 sharks in 2015 (Fig 7). The numbers of retained sharks increased for both species over the 3-year period. The 2013 and 2014 estimates should be viewed as indicative only, because they relied on constant catch ratios per subfleet based on 2015 observer samples (no observer samples available for 2013 and 2014).

Fig 7. Estimated numbers of retained and discarded blue sharks and shortfin makos (±S.E.) by the pelagic longline fleet in 2013, 2014 and 2015.

Discussion

Assumptions made during this study were only partially met in most cases, with implications for numerical estimates and the interpretation of results. For the fleet stratification, it was assumed that fishing behaviour differed among vessels, and that the composition of landings could be used to group vessels with similar behaviour. Van Helmond et al. [35] showed that individual vessels or subfleets operating within the same management system (same rules) adopted distinct fishing behaviours, which resulted in different outcomes, or landings composition. In our study, distinct subfleets or fishing behaviour could be inferred from differences in landings composition (see also [17]), were consistent over the 3-year study period, and matched preferred fishing areas in posterior analysis. We did not attempt to refine subfleets further, by including vessel characteristics, differences in gear configuration, or seasonal- and marketing dynamics (see [36, 37]).

The assumption that observer samples accurately represented discard behavior of individual subfleets could only be partially met. All six vessels of subfleet 1 were sampled at least once, and one of them was sampled twice during 2015. Samples were spread nearly evenly across quarter years, to reduce seasonal bias in estimates (Table 1). For this subfleet, numerical estimates matched benchmark values well, implying that discard ratios could accurately be determined from samples. The other three subfleets were sampled less extensively, resulting in much higher variability of discard ratios and an increased likelihood that atypical fishing behaviour during a sampling trip or seasonality would bias discard ratios. We therefore had less confidence in discard mortality estimates determined for subfleets 2 to 4.

We estimated that the South African-flagged pelagic longline fishery caught ~150 000 blue sharks and 50 000 shortfin makos during 2015. Of these, some 24 000 blue sharks and <1 000 makos were released in a good physical condition. The combined fishing mortality (retained

and discarded dead or in a poor condition) estimate of 175 000 sharks was nearly an order of magnitude greater than average mortalities of 16 000–22 000 sharks/year caused by the same fleet in 1998–2005 [24]. An increase in fishing effort from 0.45 million hooks set in 2000 to 1.7 million hooks set in 2015 (nearly 4 times more; [10]) could partially explain the increase in shark fishing mortality. Both studies extrapolated observer-based counts to total fishing effort, although fleet stratification and estimation methods differed. But more importantly, the two studies focussed on fundamentally dissimilar time periods regarding the importance of sharks on international markets, illustrated by a 10-fold increase in reported shark landings between 2003 and 2005 [10] to benefit from increased market prices during that period [38, 39]. Despite differences in estimation methods used in the two studies, the steep increase in blue shark and shortfin mako fishing mortality over the past two decades is alarming.

Long term trends in landings data and CPUE indices support the finding of a steep increase in shortfin mako fishing mortality [10]. Shortfin mako landings reported by the South African-flagged fleet increased from 869 sharks in 2000 to 37 946 in 2015, although the earlier landings may have been under-reported [24]. The increased landings originated mostly from expanded fishing grounds over the Agulhas Bank (South area) where makos were more abundant [40, 41]. Sharply rising CPUE indices after 2004 confirmed increased targeting of makos [10]. Our field samples showed that overall, only 4% of captured makos were discarded in 2015, hence landings data closely approximated fishing mortality, with discard mortalities contributing little.

Sporadic targeting of blue sharks, inferred from CPUE peaks and increased landings in some years [10] could partially explain the increase in blue shark fishing mortality. Nevertheless, the bulk of blue shark fishing mortality resulted from discards of captured sharks. Some 68% of captured blue sharks were discarded as unwanted catch during observer sampling in 2015,

mostly by subfleets 1 and 2 (tuna and swordfish directed). Extrapolations from observer data suggested that of 36 000 blue sharks captured by subfleet 1, 96% were discarded, of which 35% were dead or in poor condition. Of 68 000 blue sharks captured by subfleet 2, 81% were discarded, nearly all dead or in poor condition. The other two subfleets landed mainly sharks and therefore retained most blue sharks captured (est. 90% of 26 000 sharks caught by subfleet 3; 97% of 8 000 sharks by subfleet 4). Overall, selective fishing for tunas and swordfish contributed most to blue shark fishing mortality, because vessels discarded large numbers of sharks.

Most discarded blue sharks were either dead (58%) or in poor physical condition (4%). Our estimates were based on observations at discarding, and include mortalities resulting from the capture process (at-vessel-mortality or AVM) combined with those suffered during onboard handling, such as clubbing, cutting to remove hooks, long air-exposure times, or a combination of factors. Considering that published AVM values for blue sharks is generally <25% (reviewed by Ellis et al. [4]), on-board handling appears to have contributed substantially to discard mortalities in the present study. We do not rule out the possibility that blue sharks are purposefully killed before discarding on some vessels, to prevent them from predating and damaging hooked fish after release [13]. The elevated blue shark mortalities may alternatively reflect fishing in nursery areas (G. Jordaan, pers. observation; [42]) where neonate or small juvenile blue sharks are presumably less likely to survive capture and handling than larger sharks.

Some 82% of shortfin makos in field samples were dead when discarded. The very high rate compared to published AVM of 5–56% [4] was expected, because only badly damaged shortfin makos (depredated, decomposed) were discarded – the rest were kept (96% of catches) because of their comparatively high market value. Several other pelagic sharks were discarded by the

fishery, because their retention onboard is legally prohibited [26]. Most silky sharks (67%), threshers (67%), porbeagle sharks (54%) and bronze whalers (48%) were dead when discarded. Our estimates moderately exceeded published AVMs of silky- (56–66%), thresher (51–59%) and porbeagle sharks (21–44%) [4]. Oceanic whitetip and crocodile sharks were more hardy, and our estimates of 25% and 9%, respectively, compared well with published AVM ranges of 26–60% and 9–13% [4]. High variability in discard mortality rates is typical in pelagic longline fisheries, because time spent hooked, line configuration, hook type, handling practices, air-exposure time, and shark species, size or sex can all affect mortality rates [3-5, 13].

Subfleet fishing strategies were strongly influenced by spatial considerations. Vessels fishing selectively for tunas and swordfish operated in the East, West and Southwest areas, and those landing primarily sharks frequented the South (makos) and Southwest (blue sharks and makos) (Table 2 and [10]). Studies from other ocean regions confirm that the spatial distribution of fishing effort determines the species composition of catches made by pelagic longlines [13, 43-46]. Hotspots of blue shark and shortfin mako abundance in the South and Southwest areas include blue shark nursery areas in the Benguela/Agulhas Current confluence [42, 43] and feeding grounds for juvenile shortfin makos near the Agulhas Bank edge in winter and spring [40]. The spatial and temporal dynamics of blue sharks and shortfin makos off South Africa, particularly blue shark nursery grounds, suggest that dynamic (event-triggered) spatio-temporal closures [47-49] can be considered as a fisheries management option to reduce blue shark discard mortalities in vessels targeting tunas and swordfish.

Our study has several implications for the management of the South African-flagged pelagic longline fishery. Most pressing is the steep increase in fishing mortality of blue sharks and shortfin makos. Makos remained a primary target species of parts of the fleet, despite their official status as bycatch since 2005. A permit condition restricting shark landings to 60% of

landed total mass in any quarter [50] is unlikely to succeed in changing fisher behaviour (see [37]), because it is set too high to be effective. Shortfin makos have recently (2019) been listed on CITES Appendix II [51] which will place stricter controls on international trade. The listing provides a clear incentive for reviewing mako mortalities and adjusting upper catch limits. Alternatively, makos can be managed as a primary fisheries resource, subject to sustainable management objectives.

The bulk of blue shark mortalities resulted from discards by vessels fishing selectively for tunas and swordfish. Blue shark discard mortality rates were twice as high as published AVMs [4, 52] suggesting that onboard handling contributed substantially to discard mortalities. The prohibition of wire leaders on hooks [53] aims to reduce the incidence of sharks brought onboard, thus obviating the need to handle them. Zollett and Swimmer et al. [54] suggested that routine training of fishermen on handling practices and the consequences of removing apex predators would reduce blue shark discard mortalities.

To conclude, stratification of the pelagic longline fleet based on landings composition (a proxy for fishing behaviour) provided a well-supported framework for observer-based sampling of discard ratios and estimation of unreported shark mortalities. Numerical estimates indicated a near 10-fold increase in shark fishing mortality between 2000 and 2015, in agreement with upwards trends in fishing effort, reported landings and recent CPUE estimates [10]. Escalating shortfin mako fishing mortality was attributed to increased targeting to supply higher market demand. Discarding of blue sharks by selective fishing for tunas and swordfish had a greater impact on their fishing mortality than retention by shark-directed subfleets. Higher levels of observer sampling are required to increase confidence in discard ratio estimates.

Acknowledgements

We thank CapFish, particularly Sihle Victor Ngcongo and Willem Louw, for their advice on how the pelagic longline fishing fleet operates and their efforts in organising the observer sampling trips for the first author (G.L Jordaan). We thank the skippers and crew of the longline vessels for providing insights into various fishing strategies and techniques. Thanks are due to DEFF, particularly Charlene da Silva and Wendy West, for providing the landings and logbook data for the study, and for their advice.

References

1. Campana SE. Transboundary movements, unmonitored fishing mortality, and ineffective international fisheries management pose risks for pelagic sharks in the Northwest Atlantic. Canadian journal of fisheries and aquatic sciences. 2016;73(10):1599-607. doi: 10.1139/cjfas-2015-0502.

2. Gilman E, Suuronen P, Chaloupka M. Discards by global tuna fisheries. Marine Ecology Progress Series. 2017;582. doi: 10.3354/meps12340.

3. Campana SE, Joyce W, Fowler M, Showell M. Discards, hooking, and post-release mortality of porbeagle (*Lamna nasus*), shortfin mako (*Isurus oxyrinchus*), and blue shark (*Prionace glauca*) in the Canadian pelagic longline fishery. ICES Journal of Marine Science. 2016;73(2):520-8.

4. Ellis JR, McCully Phillips SR, Poisson F. A review of capture and post-release mortality of elasmobranchs. Journal of Fish Biology. 2017;90(3):653-722. doi: 10.1111/jfb.13197.

5. Gallagher A, Orbesen E, Hammerschlag N, Serafy J. Vulnerability of oceanic sharks as pelagic longline bycatch. Global Ecology and Conservation. 2014;1:50-9.

6. Tolotti MT, Filmalter JD, Bach P, Travassos P, Seret B, Dagorn L. Banning is not enough: The complexities of oceanic shark management by tuna regional fisheries management organizations. Global Ecology and Conservation. 2015;4:1-7. doi: http://dx.doi.org/10.1016/j.gecco.2015.05.003.

7. Oliver S, Braccini M, Newman SJ, Harvey ES. Global patterns in the bycatch of sharks and rays. Marine Policy. 2015;54:86-97. doi: http://dx.doi.org/10.1016/j.marpol.2014.12.017.

8. Clarke SC, Francis MP, Griggs LH. Review of shark meat markets, discard mortality and pelagic shark data availability, and a proposal for a shark indicator analysis. Wellington, New Zealand: Ministry for Primary Industries, 2013 ISSN 1179-5352.

9. Fields AT, Fischer GA, Shea SKH, Zhang H, Abercrombie DL, Feldheim KA, et al. Species composition of the international shark fin trade assessed through a retail-market survey in Hong Kong. Conservation Biology. 2018;32(2):376-89. doi: 10.1111/cobi.13043.

10. Jordaan GL, Santos J, Groeneveld JC. Effects of inconsistent reporting, regulation changes and market demand on abundance indices of sharks caught by pelagic longliners off southern Africa. PeerJ. 2018;6:e5726. doi: 10.7717/peerj.5726. PubMed PMID: 30386693.

11. Davidson LN, Krawchuk MA, Dulvy NK. Why have global shark and ray landings declined: improved management or overfishing? Fish and Fisheries. 2016;17(2):438-58.

12. Watson J, Kerstetter D. Pelagic longline fishing gear: a brief history and review of research efforts to improve selectivity. Marine Technology Society Journal. 2006;40(3):6-11.

13. Gilman E, Clarke S, Brothers N, Alfaro-Shigueto J, Mandelman J, Mangel J, et al. Shark interactions in pelagic longline fisheries. Marine Policy. 2008;32(1):1-18. doi: 10.1016/j.marpol.2007.05.001.

14. Ward P, Lawrence E, Darbyshire R, Hindmarsh S. Large-scale experiment shows that nylon leaders reduce shark bycatch and benefit pelagic longline fishers. Fisheries Research. 2008;90(1–3):100-8. doi: http://dx.doi.org/10.1016/j.fishres.2007.09.034.

15. Coelho R, Santos MN, Amorim S. Effects of hook and bait on targeted and bycatch fishes in an Equatorial Atlantic pelagic longline fishery. Bulletin of Marine Science. 2012;88(3):449-67.

16. Kumar KVA, Pravin P, Meenakumari B. Bait, Bait Loss, and Depredation in Pelagic Longline Fisheries–A Review. Reviews in Fisheries Science & Aquaculture. 2016;24(4):295-304. doi: 10.1080/23308249.2016.1162134.

17. He X, Bigelow KA, Boggs CH. Cluster analysis of longline sets and fishing strategies within the Hawaii-based fishery. Fisheries Research. 1997;31(1):147-58. doi: http://dx.doi.org/10.1016/S0165-7836(96)00564-4.

18. James KC, Lewison RL, Dillingham PW, Curtis KA, Moore JE. Drivers of retention and discards of elasmobranch non-target catch. Environmental Conservation. 2016;43(1):3-12. doi: 10.1017/s0376892915000168.

19. da Silva C, Booth AJ, Dudley SFJ, Kerwath SE, Lamberth SJ, Leslie RW, et al. The current status and management of South Africa's chondrichthyan fisheries. African Journal of Marine Science. 2015;37(2):233-48. doi: 10.2989/1814232x.2015.1044471.

20. Dulvy NK, Baum JK, Clarke S, Compagno LJ, Cortés E, Domingo A, et al. You can swim but you can't hide: the global status and conservation of oceanic pelagic sharks and rays. Aquatic Conservation: Marine and Freshwater Ecosystems. 2008;18(5):459-82.

21. Musick JA. Criteria to define extinction risk in marine fishes: the American Fisheries Society initiative. Fisheries. 1999;24(12):6-14.

22. Block BA, Jonsen ID, Jorgensen SJ, Winship AJ, Shaffer SA, Bograd SJ, et al. Tracking apex marine predator movements in a dynamic ocean. Nature. 2011;475(7354):86.

23. Kohler NE, Turner PA, Hoey JJ, Natanson LJ, Briggs R. Tag and recapture data for three pelagic shark species: blue shark (*Prionace glauca*), shortfin mako (*Isurus oxyrinchus*), and porbeagle (*Lamna nasus*) in the North Atlantic Ocean. Collective Volume of Scientific Papers - ICCAT. 2002;54(4):1231-60.

24. Petersen S, Honig M, Ryan P, Underhill L, Compagno LJ. Pelagic shark bycatch in the tuna-and swordfish-directed longline fishery off southern Africa. African Journal of Marine Science. 2009;31(2):215-25.

25. DEFF. National Plan of Action for the Conservation and Management of Sharks (NPOA-Sharks). Cape Town, South Africa: Department of Environment, Forestry and Fisheries, 2013 IOTC-2013-SC16-INF15.

26. da Silva C, Winker H, Parker D, Wilke C, Lamberth SJ, Kerwath S. Update and review of the NPOA for Sharks South Africa. Indian Ocean Tuna Commission, 2018 IOTC-2018-WPEB14-11_Rev1.

27. R_Development_Core_Team. R: A language and environment for statistical computing. Vienna, Austria.: R Foundation for Statistical Computing; 2020.

28. Müllner D. Fast Hierarchical Clustering Routines for R and 'Python' – Version 1.1.25: Package ‘fastcluster’. CRAN Repository, 2018.

29. NCSS. Hierarchical Clustering / Dendrograms. NCSS Statistical Software - NCSS Documentation. Kaysville, Utah, USA: NCSS Statistical Software; 2018.

30. Maechler M, Rousseeuw PJ, Struyf A, Hubert M, Hornik K, Studer M, et al. ``Finding Groups in Data'': Cluster Analysis Extended Rousseeuw et al. – Version 2.1.0: Package ‘cluster’. CRAN Repository, 2019.

31. Kaufman LR, Rousseeuw PJ. Finding groups in data: An introduction to cluster analysis. Hoboken, New Jersey John Wiley & Sons Inc.; 1990. 725 p.

32. Kassambara A, Mundt F. Extract and Visualize the Results of Multivariate Data Analyses – Version 1.0.7: Package ‘factoextra’. CRAN Repository, 2020.

33. FAO. On Board Guide for the Identification of Pelagic Sharks and Rays of the Western Indian Ocean. Rome, Italy: Food and Agriculture Organisation of the United Nations, 2014.

34. Conquest L, Burr R, R. D, Chavarria JB, Gallucci VF. Sampling methods for Stock Assessment of Small-Scale Fisheries in Developing Countries. In: Gallucci VF, Saila S, Gustafson DJ, Rothschild BJ, editors. Stock Assessment: Quantitative Methods and Applications for Small Scale Fisheries. Florida, U.S.A: CRC Press, Lewis Publishers; 1996.

35. Van Helmond ATM, Chen C, Trapman BK, Kraan M, Poos JJ. Changes in fishing behaviour of two fleets under fully documented catch quota management: Same rules, different outcomes. Marine Policy. 2016;67:118-29. doi: https://doi.org/10.1016/j.marpol.2016.01.029.

36. Davie S, Lordan C. Definition, dynamics and stability of métiers in the Irish otter trawl fleet. Fisheries Research. 2011;111(3):145-58. doi: https://doi.org/10.1016/j.fishres.2011.07.005.

37. Wise L, Murta AG, Carvalho JP, Mesquita M. Qualitative modelling of fishermen's behaviour in a pelagic fishery. Ecological Modelling. 2012;228:112-22.

38. Autoridad_Portuaria_de_Vigo. Puerto de Vigo - Annual Reports (2003 - 2015) VIGO (Pontevedra): Puertos del Estado; 2016 [2016/10/13]. Available from: http://www.apvigo.com/control.php?sph=a_iap=1251%%p_rpp=1%%s_idm=3.

39. Dent F, Clarke S. State of the global market for shark products. Rome, Italy: Food and Agriculture Organisation of the United Nations, 2015 FAO Fisheries and Aquaculture Technical Paper No. 590.

40. Groeneveld JC, Cliff G, Dudley S, Foulis A, Santos J, Wintner S. Population structure and biology of shortfin mako, *Isurus oxyrinchus*, in the south-west Indian Ocean. Marine and Freshwater Research. 2014;65(12):1045-58.

41. Smith C. National Report of South Africa. Cape Town: Indian Ocean Tuna Commission, 2005.

42. da Silva C, Kerwath S, Wilke C, Meyer M, Lamberth S. First documented southern transatlantic migration of a blue shark *Prionace glauca* tagged off South Africa. African Journal of Marine Science. 2010;32(3):639-42.

43. Coelho R, Mejuto J, Domingo A, Yokawa K, Liu K-M, Cortés E, et al. Distribution patterns and population structure of the blue shark (*Prionace glauca*) in the Atlantic and Indian Oceans. Fish and Fisheries. 2018;19(1):90-106. doi: 10.1111/faf.12238.

44. Mas F, Forselledo R, Domingo A. Catch disposition of blue sharks (*Prionace glauca*) caught by longliners in the Southwestern Atlantic. Collective Volume of Scientific Papers - ICCAT. 2016;72(5):1113-23.

45. Megalofonou P, Yannopoulos C, Damalas D. Incidental catch and estimated discards of pelagic sharks from the swordfish and tuna fisheries in the Mediterranean Sea. Fisheries Bulletin. 2005;103:620-34.

46. Mejuto J, Garcia-Cortés B, Ramos-Cartelle A, De la Serna JM, González-González I, Fernández-Costa L. Standardized catch rates for the blue shark (*Prionace glauca*) and shortfin mako (*Isurus oxyrinchus*) caught by the Spanish surface longline fleet in the Atlantic Ocean during the period 1990-2007. Collective Volume of Scientific Papers – ICCAT. 2009;64(5):1509-21.

47. Dunn DC, Boustany AM, Halpin PN. Spatio-temporal management of fisheries to reduce by-catch and increase fishing selectivity. Fish and Fisheries. 2011;12(1):110-9. doi: 10.1111/j.1467-2979.2010.00388.x.

48. Grantham HS, Petersen SL, Possingham HP. Reducing bycatch in the South African pelagic longline fishery: the utility of different approaches to fisheries closures. Endangered Species Research. 2008;5(2-3):291-9.

49. Kinney MJ, Simpfendorfer CA. Reassessing the value of nursery areas to shark conservation and management. Conservation Letters. 2009;2(2):53-60. doi: 10.1111/j.1755-263X.2008.00046.x.

50. DEFF. Permit Conditions: Large Pelagic Longline Fishery. Fishing Season: 2020/2021. Cape Town, South Africa: Department of Environment, Forestry and Fisheries, 2020.

51. CITES. Notes on the Proposal for Inclusion of the Shortfin Mako (*Isurus oxyrinchus*) and Longfin Mako (*Isurus paucus*) Sharks in Appendix II of Cites in Relation to the Stock Status and Scientific Advice Provided to the ICAAT Commission. Eighteenth meeting of the Conference of the Parties Geneva (Switzerland), 17-28 August 2019: Convention on International Trade in Endangered Species of Wild Fauna and Flora, 2019 Report No. CoP18 Inf. 8.

52. Campana SE, Joyce W, Manning MJ. Bycatch and discard mortality in commercially caught blue sharks *Prionace glauca* assessed using archival satellite pop-up tags. Marine Ecology Progress Series. 2009;387:241-53.

53. DEFF. Permit Conditions: Large Pelagic Longline Fishery. Fishing Season: 2018/2019. Cape Town, South Africa: Department of Environment, Forestry and Fisheries, 2018.

54. Zollett EA, Swimmer Y. Safe handling practices to increase post-capture survival of cetaceans, sea turtles, seabirds, sharks, and billfish in tuna fisheries. Endangered Species Research. 2019;38:115-25.

Supporting information

S1 Table. The numbers of blue and shortfin mako sharks landed per subfleet in 2015 compared to the estimates of retained sharks, those discarded in good and poor condition, respectively including the standard error (S.E.).

S1 Fig. Dendrogram of vessels clustered into four subfleets based on the species composition of landings in 2015. Vessels shown in bold were sampled by observers. Subfleet 1 (red) landed mainly tunas and swordfish; Subfleet 2 (green) landed tunas, swordfish and sharks; Subfleet 3 (purple) landed shortfin mako and blue sharks; Subfleet 4 (yellow) landed mainly shortfin makos.

S2 Fig. Dendrogram of vessels clustered into four subfleets based on the species composition of landings in 2014. Vessels shown in bold were sampled by observers. Subfleet 1 (red) landed mainly tunas and swordfish; Subfleet 2 (green) landed tunas, swordfish and sharks; Subfleet 3 (purple) landed shortfin mako and blue sharks; Subfleet 4 (yellow) landed mainly shortfin makos.

S3 Fig. Dendrogram of vessels clustered into four subfleets based on the species composition of landings in 2013. Vessels shown in bold were sampled by observers. Subfleet 1 (red) landed mainly tunas and swordfish; Subfleet 2 (green) landed tunas, swordfish and sharks; Subfleet 3 (purple) landed shortfin mako and blue sharks; Subfleet 4 (yellow) landed mainly shortfin makos.

Fig1

Click here to download Figure Fig1.tif

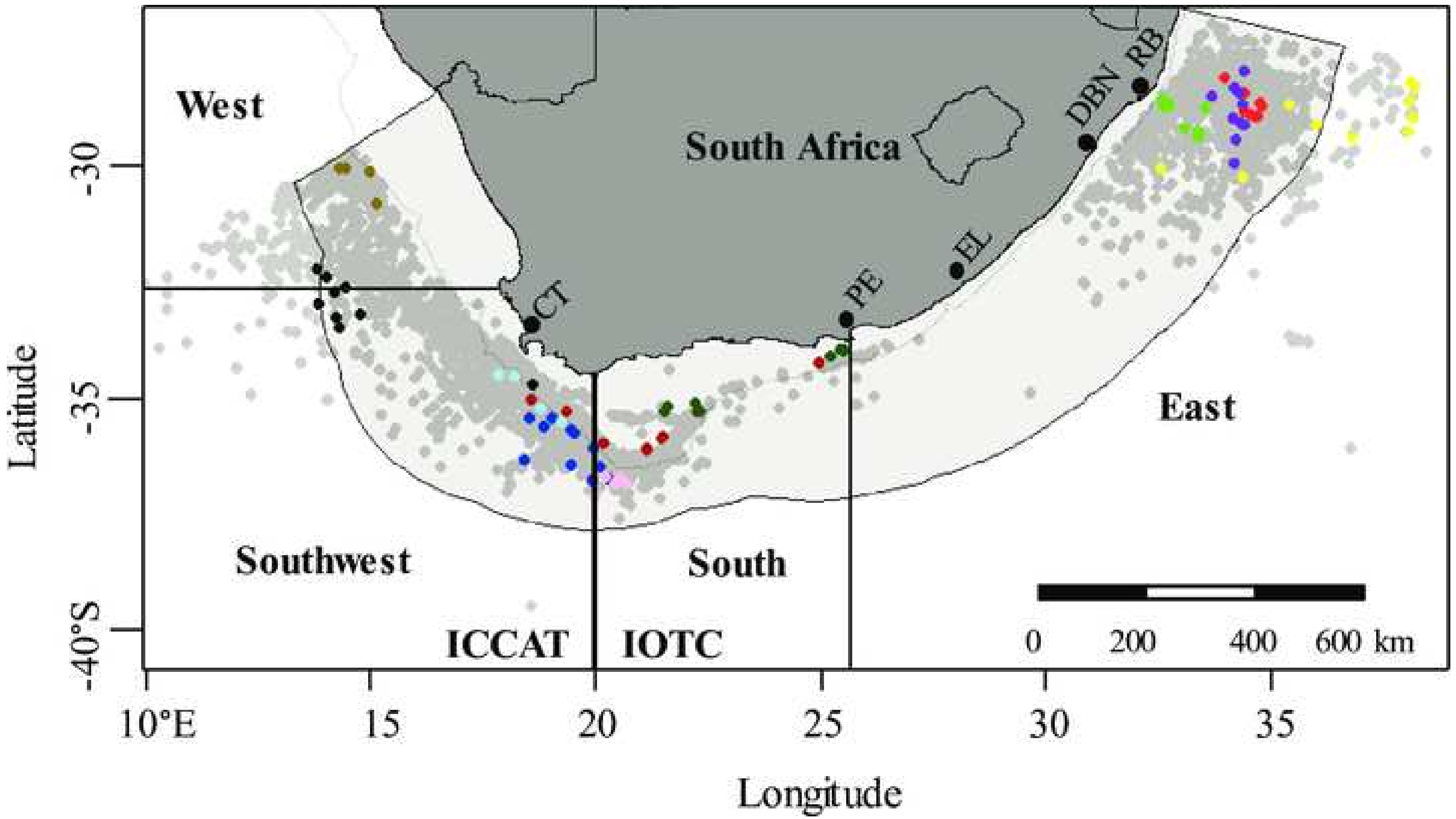

Fig2

Click here to download Figure Fig2.tif

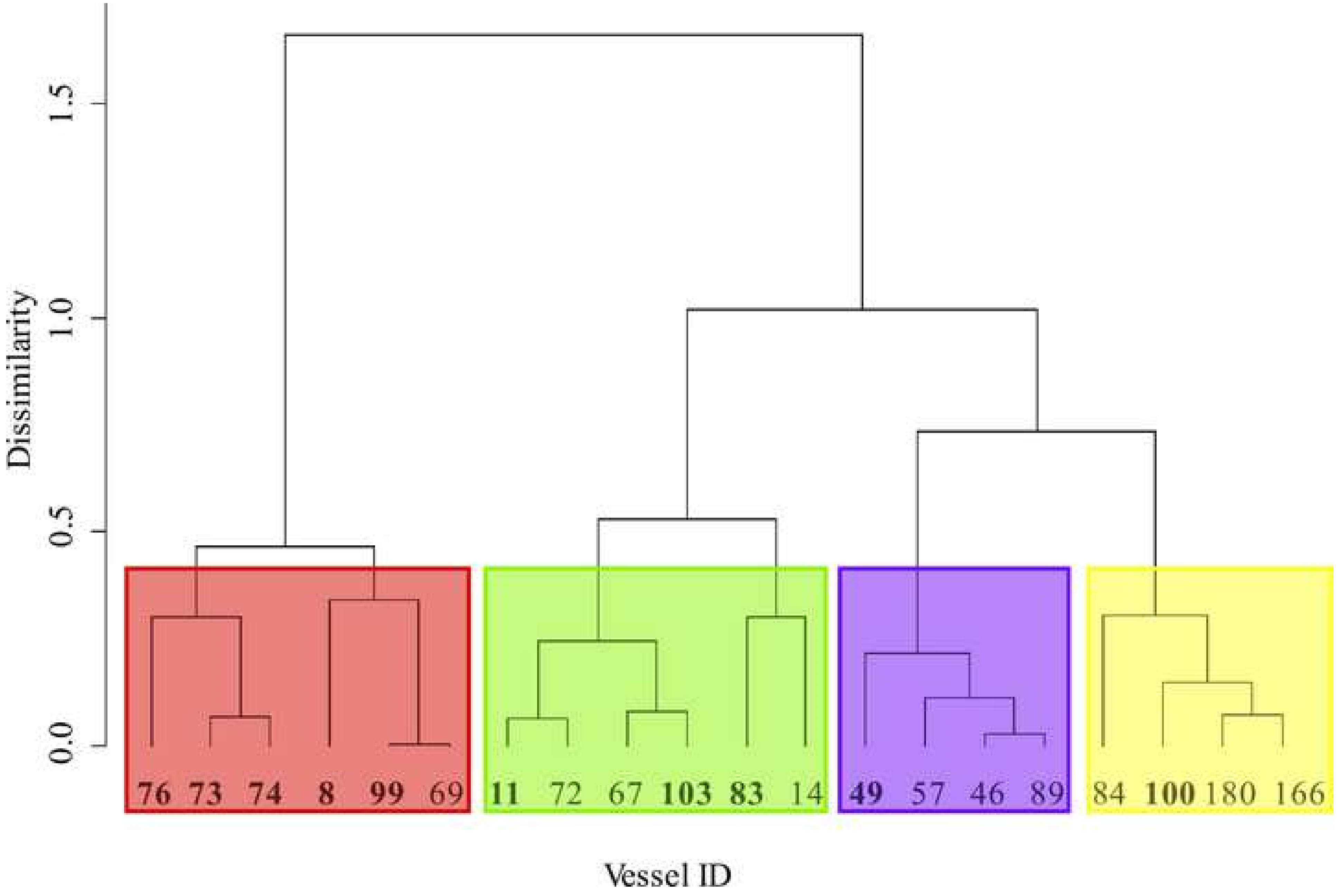

Fig3

Click here to download Figure Fig3.tif

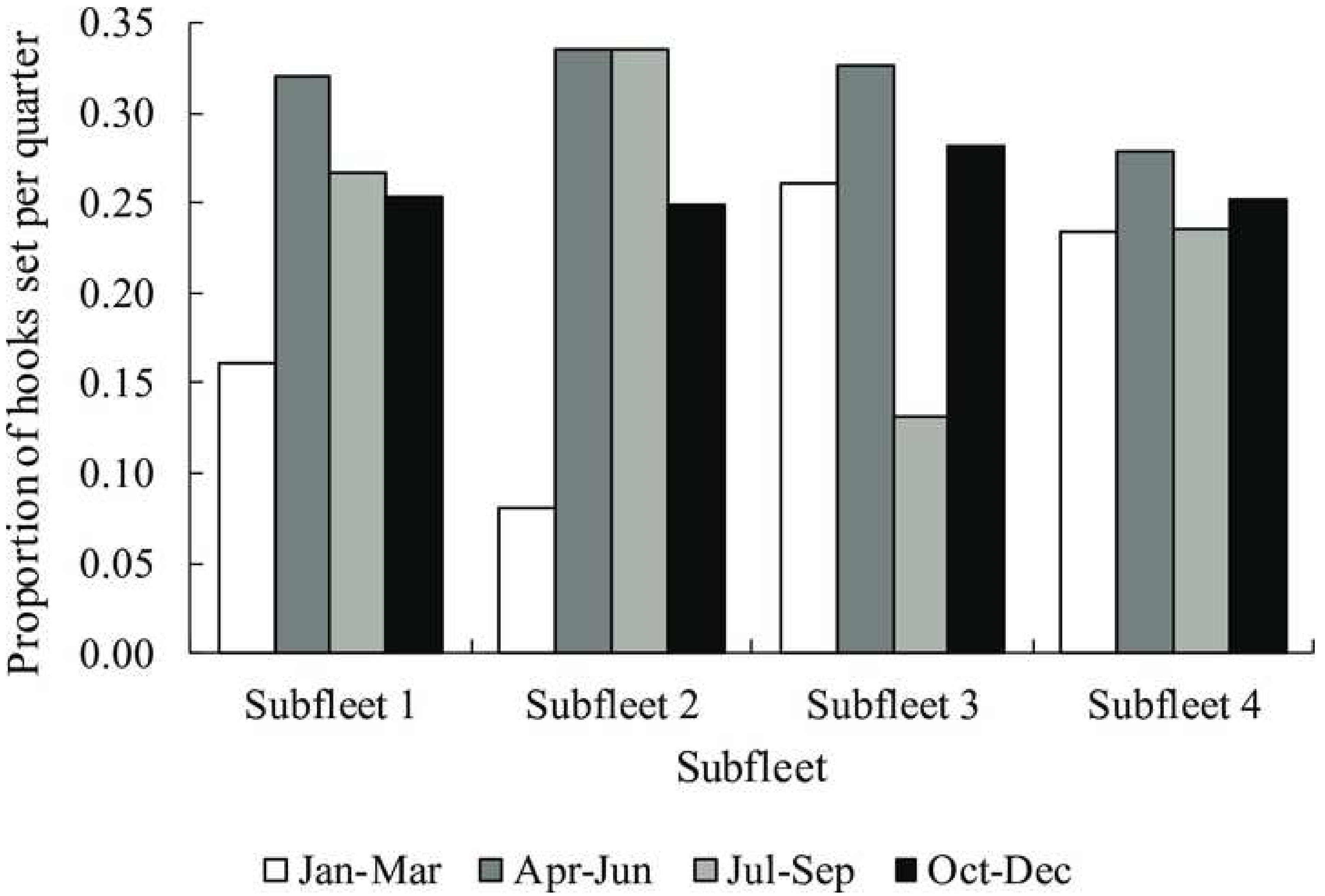

Fig4

Click here to download Figure Fig4.tif

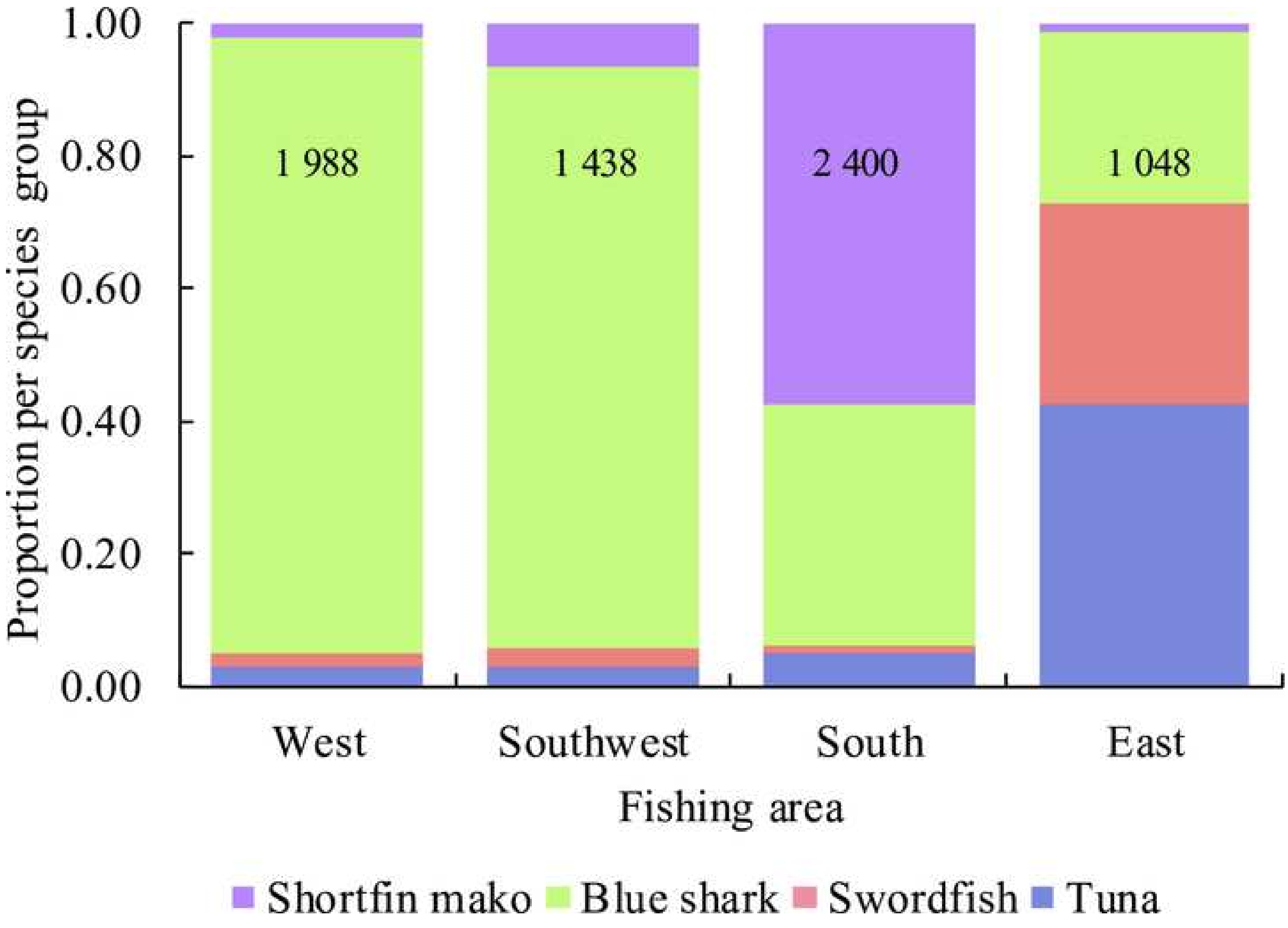

Fig5 Click here to download Figure Fig5.tif

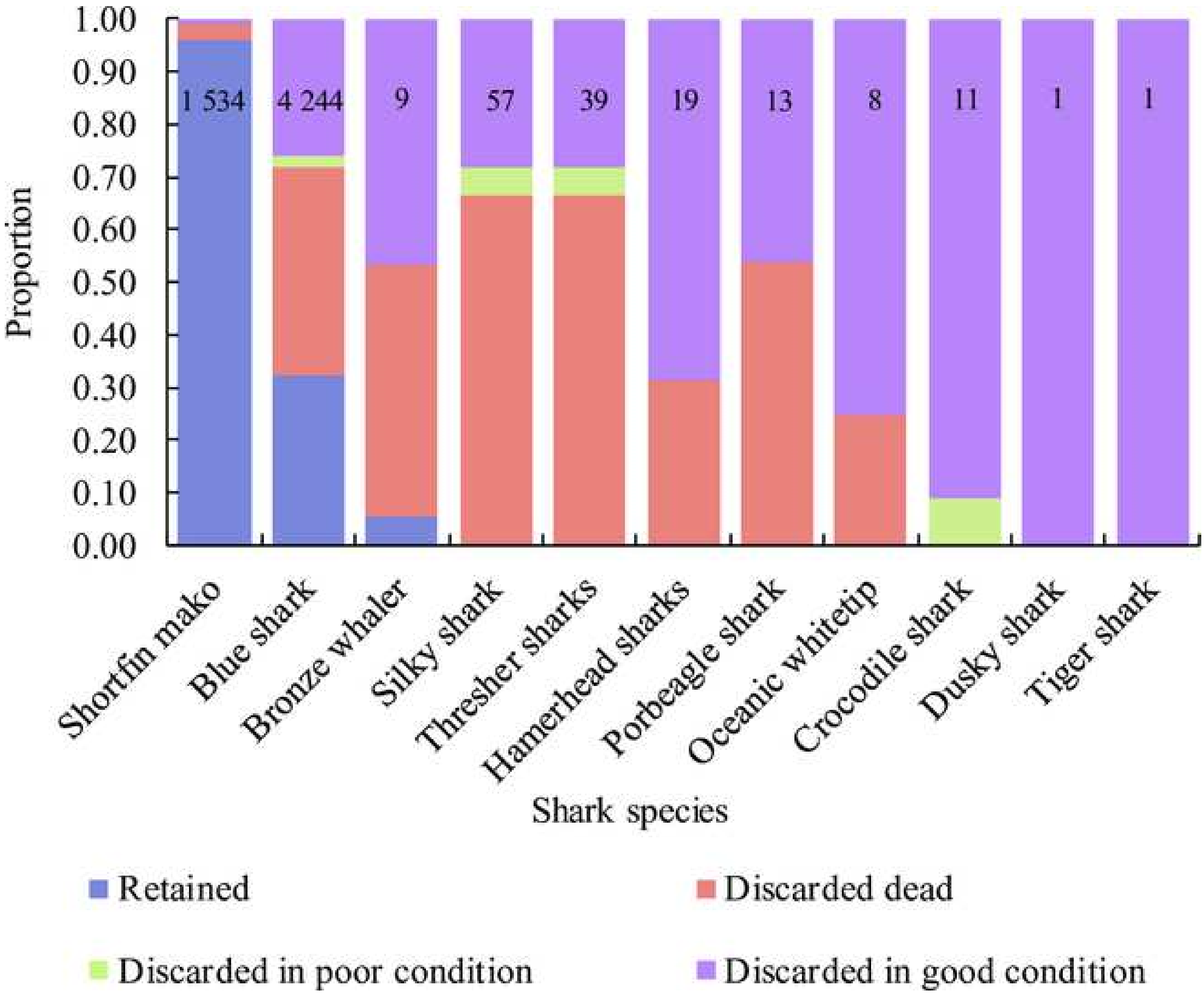

Click here to download Figure Fig6.tif

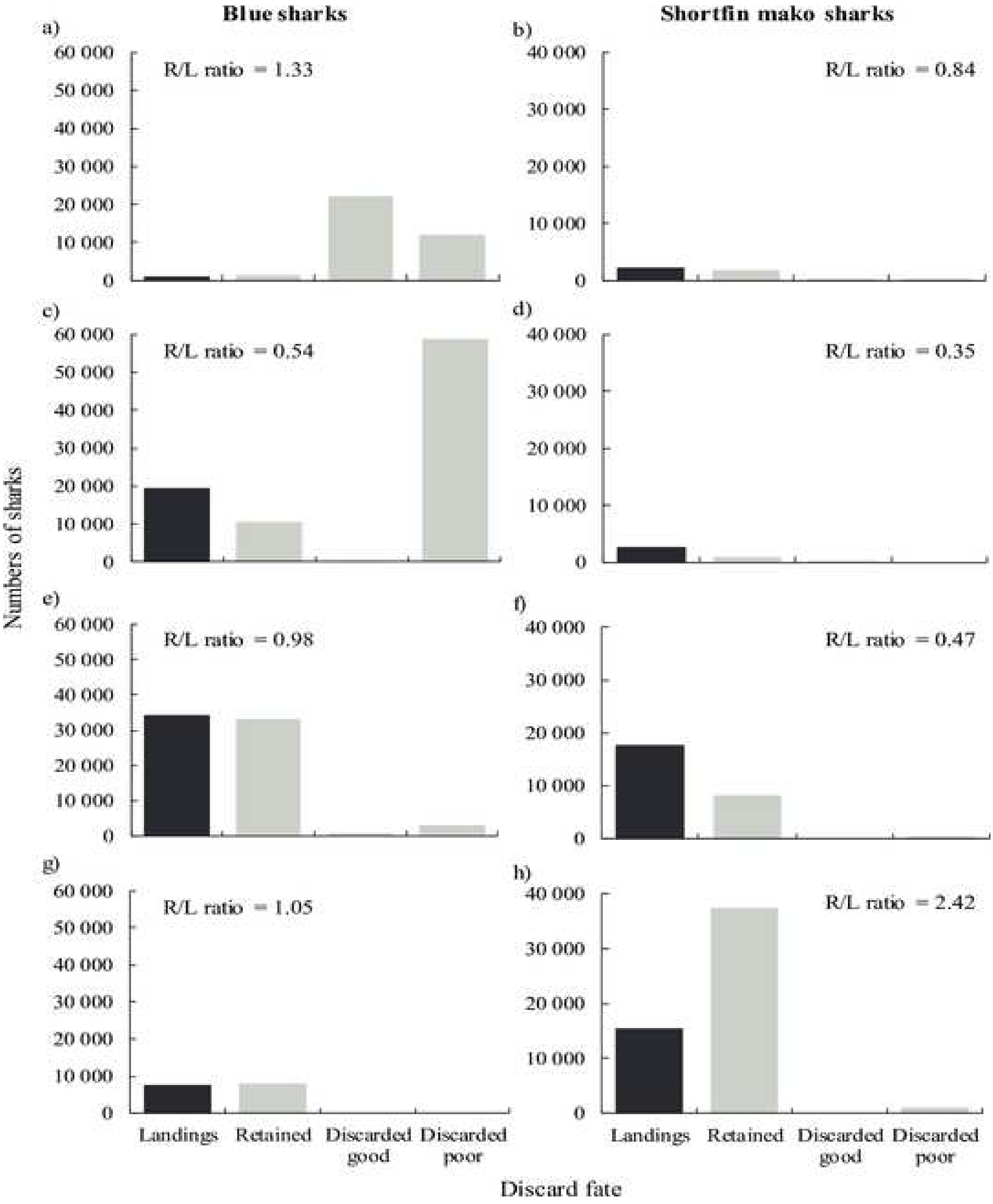

Fig7

Click here to download Figure Fig7.tif

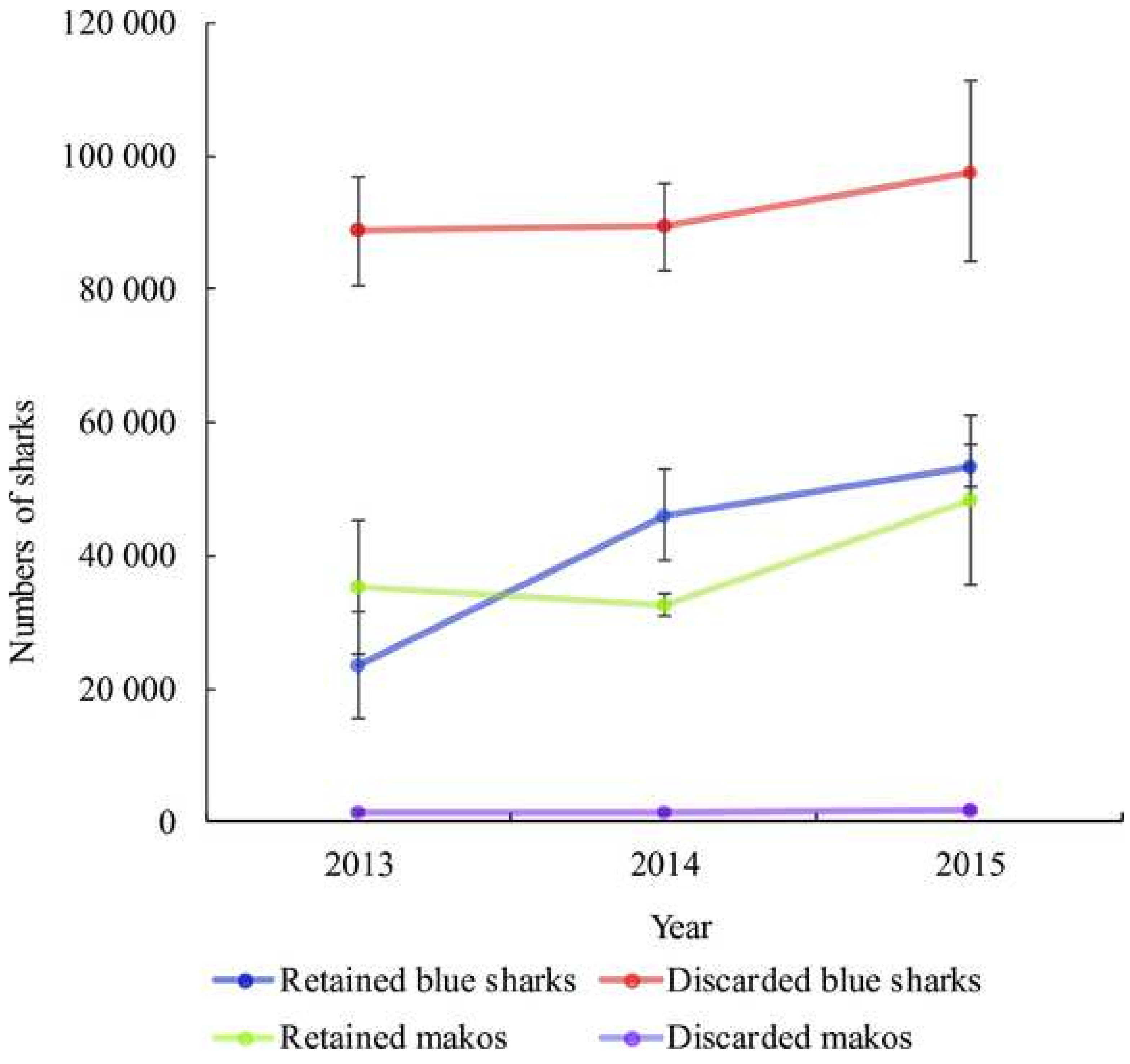

S1_Fig

Click here to access/download
Supporting Information
S1_Fig.tif

S2_Fig

Click here to access/download
Supporting Information
S2_Fig.tif

S3_Fig

Click here to access/download
Supporting Information
S3_Fig.tif

S1 Table

Click here to access/download
Supporting Information
S1 Table.docx

Click here to access/download
Supporting Information
Observer_data_Plos_One.xlsx

Click here to access/download
Supporting Information
Landings_Data_2013_2015_Plos_One.xlsx
