# Supplementary material for: Shark discards in selective and mixed-species pelagic longline fisheries
Source: PLoS One. 2020 Aug 31;15(8):e0238595. doi: 10.1371/journal.pone.0238595 (PMC7458300; doi:10.1371/journal.pone.0238595)
Supplement: Attachment — Submitted filename: PLoS editorial comment_Shark discards.docx [file pone.0238595.s008.docx]

**PLoS editorial comment_Shark discards (PONE-D-20-12467)**

Interesting study. I think the text needs a bit of work as there are some confusing statements and some grammatical errors. The authors introduce a lot of abbreviations, but it is PLoS formatting that abbreviations be used at least three times in the text or not introduced. The manuscript also lacks an ethics statement in the methods detailing all the permits and permissions needed to undertake the study. This study may not have required any, but that should be clear. Please see the online submission guidelines for more information

Line 24: Sentence a bit awkward as written and ‘several species’ is a bit vague and really a bit of an understatement. I would rewrite.

Lines 30-31: Should provide the scientific names here as well

Line 35: I don’t think this is grammatically correct and should be ‘which was comprised of’

Line 60: I wonder if ‘discounts’ is the right word here. Maybe say ‘because it does not include discard mortalities’

Line 69: Need to provide the scientific names here in the main text as well

Line 82: Should be ‘life-history traits’

Line 85: Should be ‘high-seas fishing fleets’

Line 87: CITES needs to be spelled out here and do not introduce the abbreviation CITES as it is only used once more. It is PLoS formatting that abbreviations should be used at least three times or not introduced.

Line 92: Delete the hyphen after ‘catch’ as it is not required

Line 96: This is confusing. The authors introduce these abbreviations but don’t use them again. Is this ‘southwest’ the same as is being referred to on line 119? Seems like they may be different things. If so please clarify in the text and do not introduce the abbreviations if they are not used at least 3 more times.

Lines 101 and 102: Spell out ICCAT and IOTC and do not introduce the abbreviations.

Line 102: I am not sure what ‘respectively’ is supposed to refer to here but it doesn’t make sense as written so please delete.

Line 107: Confusing as written ‘in a National Plan of Action’. I am assuming this should be ‘in the National Plan of Action for South Africa’? Otherwise it seems very random

Line 108: Delete ‘NPOA’ as it is not used at least three times.

Line 119: Again, is this the same as the SW introduced on line 96?

Line 131: Again, spell out IOTC and ICCAT

Line 132: Do not introduce the abbreviation ‘EEZ’

Line 137: This should be ‘were comprised of’

Line 197: I am not sure what ‘respectively’ is supposed to refer to here but it doesn’t make sense as written so please delete.

Line 219: ‘recovered’ sounds strange here, I would say ‘identified’ instead

Line 224: ‘recovered’ sounds strange here, I would say ‘identified’ instead

Line 225: Delete ‘supplemental data’ as it is not correct (PLoS has Supporting Information) and is also not necessary

Lines 234-235: Why is this bit about Bessel 83 important?

Line 246: Write as ‘There was considerable overlap

Line 254: Need ‘respectively’ after ‘of the hooks set’

Line 256: Need a comma before ‘but’

Line 273: I am not sure what ‘respectively’ is supposed to refer to here but it doesn’t make sense as written so please delete.

Line 281: I would say ‘In contrast’

Line 291: Delete ‘of’ before ‘nine species’

Line 306: Delete the hyphen after ‘silky’

Line 311: Delete the hyphen after ‘dusky’

Line 312: I am not sure what ‘respectively’ is supposed to refer to here but it doesn’t make sense as written so please delete.

Line 336: I would delete ‘in poor physical condition’ here as it is in the next line and you don’t need it twice

Line 346: What do the different coloured bars in the figure indicate? Should be in the caption.

Line 363: Delete the comma at the end of the line

Line 391: Delete the hyphen after ‘seasonal’

Line 404: Think this should be ‘or sharks in poor condition’

Line 405: I would replace ‘caused’ by ‘incurred’

Line 408: This is confusing ‘Both studies’. Does this mean reference 10? I think this should be redrafted to be clearer.

Line 415: Should be ‘Long-term trends’

Line 425: Need a comma before ‘could’

Line 443: Should this be ‘depredating’?

Line 449: Think this should be ‘to the published’

Line 454: delete the hyphen after ‘silky’

Line 478: Need to spell out CITES

Line 487: I would use ‘fishers’ rather than ‘fishermen’
