# Supplementary material for: Shark discards in selective and mixed-species pelagic longline fisheries
Source: PLoS One. 2020 Aug 31;15(8):e0238595. doi: 10.1371/journal.pone.0238595 (PMC7458300; doi:10.1371/journal.pone.0238595)
Supplement: Attachment — Submitted filename: Jordaan_et_al._Response to Reviewers.docx [file pone.0238595.s009.docx]

**Revisions made to manuscript (PONE-D-20-12467)**

Dear Dr. Patterson,

We thank you and the two reviewers for the many useful comments and suggestions, which were all considered, and helped us to improve the manuscript.

As requested, we made formatting changes to fit with PLoS One’s formatting style, and corrected the grammatical errors throughout. We responded to all the queries and comments in the text below. We addressed the overall comments for Reviewer 1, Reviewer 2 and your own Editorial comments, as well as the individual line by line comments. Each line-by-line response matches up to the comments mentioned by the reviewers and editor and refer to the line number in the original manuscript, and not the revised manuscript with track changes. As requested, we have uploaded the original manuscript, a manuscript with track changes as well as this response to the reviewer’s comments.

We trust that you will find the revised manuscript a much-improved product, suitable for acceptance by PLoS One.

We thank you and the reviewers for the many insights and advice provided.

Yours sincerely,

Gareth Jordaan (on behalf of the 3 authors)

**Reviewer 1**

**Overall comments**

**Reviewer 1:** There is significant concern over the lack of data from which the authors conducted their analyses. The time series is short, at only 3 years. It is evident from a previous publication from the same authors (Reference 10) that they have access to a 15 year time series. The reason for the exclusion of the additional 12 years is cause for concern.

**Authors:** The study is based on two datasets: observer data of shark catches and discards collected mainly by the first author while at sea on commercial fishing vessels; and official landings data (including logbook data) obtained from the fisheries department. The observer data are the crux of the study; data were collected during 11 trips at sea undertaken in 2015 (some of them up to 2 weeks long) during which 89 longline sets were sampled (71 000 hooks) and 6019 sharks were observed (all of this summarized in the text, and clearly showed in Table 1). The second dataset (landings and logbook data) were used to stratify the fleet into subfleets, and to assess spatio-temporal distribution of fishing effort – **during the period for which we had the observer data**.

It would have made no sense to collect observer data in 2015, but then rely on landings and logbook data collected 15 years ago, because targeting (i.e. subfleets) and the spatial distribution of fishing effort have changed enormously over this period (see Jordaan et al. 2018 – Reference 10).

We did use landings and logbook data for the 2013 – 2015 period to test whether subfleets operated in a consistent manner over the period of our study, and to investigate the effects of movements of individual vessel between subfleets. Note that cluster analyses were conducted for each year individually (2013, 2014 and 2015 – see Figures in supporting information) and that data were only combined over the 3 years when it was very clear that subfleets followed a consistent fishing strategy over the period.

Figure 7 back-extrapolated the 2015 observer data to 2013 and 2014 (for which subfleets operated in a consistent manner – see explanation above) and raised estimated numbers of sharks retained and discarded accordingly. Back-extrapolating any further into the past would assume that fishing strategy remained constant over time (pre-2013), and that the 2015 observer samples accurately represented discarding behaviour in the past. These assumptions were tested and rejected by Jordaan et al. 2018 (Reference 10). The reviewer’s concern, that we cherry-picked the data, is therefore fully rebutted.

Given the large amount of observer data collected by the first author (6019 sharks observed; 71 000 hooks observed), combined with official landings and logbook data for the entire fleet of 20 vessels for the 2013-2015 period (> 4 million hooks set), we contest the reviewer’s opinion that there was a “lack of data”.

**Reviewer 1:** Additionally, only one year of observer data is available, which is partial and not comprehensive of all seasons, fishing areas or vessels. Of the observed trips, only 56-80% of hooks were sampled. In three of the four subfleets, only 1 or 2 trips were covered, and therefore cannot be assumed representative of all similar trips. While a coverage rate of 5-6% for a single year is generally accepted, the lack of randomization, inclusion of all areas in all seasons and incomplete sampling effort reduces the reliability of the data substantially. Of all data collected, only Subfleet 1 could be considered robust enough for the amount of extrapolation conducted, and that is excluding the consideration that of the observed trips, only 56% of hooks were sampled.

**Authors:** One year of observer data was collected by the first author (see explanation above and Table 1 in the manuscript). The comment that “only 56 – 80% of hooks were sampled” during observed trips does not take into account the practicalities of sampling commercial fishing at sea (a 24-hour, 7 days a week operation). Indeed, 56 – 80% of hooks sampled is an exceptionally high proportion of hooks sampled by any subsampling standards.

All quarters were sampled for Subfleet 1, and we agree fully with the reviewer that Subfleet 1 is the only one for which observer data were robust enough for the extrapolations that were conducted. The assumption that observer samples accurately represented discard behaviour (i.e. data robustness) is diligently unpacked in both the results section and in the discussion of the manuscript. Data shortcomings and their effects on estimates form the central discussion points of this study, as was made clear from the very first sentence of the Abstract, i.e.: “ The conservation status of several pelagic shark species is considered vulnerable with declining populations, **yet data on shark fishing mortality remain limited** for large ocean regions.” (Revised version). The last sentence in the Abstract similarly concludes that: “Higher levels of observer sampling are required to increase confidence in discard ratio estimates”

We believe that the reviewer’s comment is fully addressed within the manuscript as it stands, at multiple levels.

**Reviewer 1:** There are multiple instances of subjective terms ("often", "nearly all", etc.) being used to categorize the data presented without supporting evidence or presenting values.

**Authors:** Unfortunately the reviewer was not specific, thus making it difficult to justify each occurrence of ‘often’ or ‘typically’ or ‘nearly all’ in the manuscript.

We do not agree that the use of ‘often’ is not justified in at least some of the cases, for example, in line 49 : “Hooked sharks often die during capture or shortly thereafter as a result of physical injuries….” Here it is exactly what we intended to say (often = frequently; many times; many cases).

We do not understand (or agree) with the reviewer’s comment (in the margin of the MS) that using often for something that occurs 35% of the time is misleading. We could not find a percentage mentioned anywhere in a search for the word “often” in online dictionaries.

**Reviewer 1:** While the authors do include acknowledgement of lack of data, bias and assumptions not being met, it is unclear why all analyses were conducted and/or data included. The recent listing of shortfin mako sharks under CITES Appendix II may play a role in this publication. It is unclear if additional observer data is available, and if so why it was not included, in addition to the exclusion of the larger time series.

**Authors:** The reviewer raises several points here. Firstly, we contend that “..the authors do include acknowledgement of lack of data etc.” is a very narrow interpretation of the study, seeing that the study actually addresses the lack of data and assumptions fully, from the first line of the abstract, right through to the end of the discussion.

Secondly, we do not understand the statement of the reviewer that it is “unclear why analyses were conducted and/or data included”. Data used and analyses done are extensively explained in the Materials and Methods section.

Thirdly, the recent listing of shortfin makos happened long after the onset of the study in 2015 – it had no influence on the decision to publish.

And fourthly, our observer data were collected according to a set protocol, to allow for this specific study. Observer data collected during other studies focussed on other objectives, and were not compatible with our objectives. Hence it was not included in this study.

**Reviewer 1:** It is the hope of the reviewer that the authors will consider expanding the time series and include all data available for analysis instead of what appears to be picking and choosing information for presenting a predetermined conclusion.

**Authors:** Expanding the time series is not possible because there is no long time-series of observer data, as explained above. We fully disagree with the reviewer’s insinuation that we ‘picked and chose information for presenting a predetermined conclusion’. The study was planned in advance with specific objectives and data protocols. The extensive text in the discussion explaining the potential influences of data limitations and assumptions made debunks the reviewer’s opinion, in this instance.

**Line by line comments (from marginal comments in pdf)**

**Line 25:** Sentence changed

**Line 39:** No change required – comment only

**Line 49:** Use of “often”. See general comments

**Line 51:** The reviewer is referred to Reference 3 – 6 for multiple examples. Too much detail for an Introduction.

**Line 89-90:** We removed the word “extremely” from the sentence and refer the reviewer to Reference 2, which based its findings on multiple tuna fisheries and an extensive literature review of discard rates in each fishery.

**Line 128-129:** Agreed. Figure title has been rephrased to make it clearer.

**Line 145:** Agreed. The weights do appear low when compared to live animals, because they are processed weights (i.e. gut removed). It is very seldom (if ever) that catch (caught for export) is landed whole without being gutted, therefore the recorded weight is not the same as live weight. We have added “(processed weight after removing the gut)”to the sentence to clarify.

**Observer sampling:** The reviewer’s concerns are addressed in the general comments

**Line 177-179:** The reviewer’s concerns over sampling strategy are addressed in the general comments. Greater variability in discard ratios for subfleets sampled less extensively are discussed in depth in the Discussion section.

**Line 220:** Twenty individual vessels landed catch between 2013 and 2015. Reference 10 (61 vessels) refers to a much longer period (2000 – 2015) which is not relevant to the present study.

**Line 225:** We have added ‘in 2015’ to the figure caption to indicate that the vessels in bold were sampled by the observers in 2015. Observers did not sample vessels in 2013 and 2014; only in 2015. See general comments.

**Line 226:** Thank you, our mistake. Changed to 15 in 2013 and 16 in 2014.

**Line 234-235:** Agreed. Sentence deleted. Vessel 83 was given as an example of a vessel that moved between subfleets in our analysis, by adopting a different fishing strategy. But in hindsight it is not needed here in the text.

**Line 237-238:** The use of data and the assumptions made are explained in detail in the general comments part above.

**Line 320-322:** Results from subfleets 3 and 4 remain important within the context of the study, despite the lower sampling coverage. For example, it highlights that a higher observer sampling effort (as seen in subfleet 1) is required when estimating discards. Furthermore, even though there may be over-estimation, it highlights the high incidence of unreported discards from this fishery.

**Lines 324-326:** No comment.

**Line 353:** The reviewer does not appreciate the realities of sampling at sea on a commercial longliner. Detailed explanation given in general comments above.

**Line 356:** See Figure 6c.

**Lines 362-364:** See comments made for lines 320-322.

**Lines 368-369:** A spatial effect is shown for specifically what this vessel landed, shortfin makos. The vessel concentrated its fishing effort in areas of high shortfin mako abundance. We elaborated in the discussion with relevant references.

**Line 381:** Explained in detail under general comments above

**Line 404-406:** All issues to do with data, bias and confidence have been rebutted in the general comments section and individual responses above. The bias and confidence inherent in the sampling are discussed in detail in the manuscript, highlighting the potential effects on estimates – i.e. we are confident in the estimates for subfleet 1, and less confident for subfleets 3 – 4, with limitations explained. Comparing results with other studies forms part of the interpretive process in science – without it any results produced by a study would have to be seen in isolation, and no progress would be made.

**Line 431:** Added in percentage.

**Lines 441-442:** This was witnessed by the observer (the first author GJ); and the shark’s condition when discarded was recorded. The study was not looking at the AVM therefore there was no need to record the state of the animal when brought on board.

**Line 473-474:** “Steep increase” is a valid statement despite data limitations for reasons mentioned above in line 381 and lines 404-406.

**Line 483-484:** See comments for lines 441-443.

**Lines 489-491:** Added in ‘especially for subfleet 1’.

**Line 492:** Change made. This was the authors mistake.

**Reviewer 2**

**Lines 24 and 25:** Agreed. Sentence changed, please see revised manuscript with track changes.

**Line 27:** Scientific name added for swordfish, genus name added for tuna.

**Line 35:** Done.

**Line 44:** Changed to fleet.

**Line 53:** Agreed. Changed the sentence as follows: ‘In most pelagic longline fisheries, discarded sharks are not reported in fisher logbooks [1], and their numbers, species composition and associated fishing mortality are therefore poorly known.’

**Line 56:** References 8 and 9 qualify the statement

**Line 62:** At vessel mortality rates are already included – within the context of the study they are either part of ‘discarded dead’ or as observed landings. No change made.

**Line 63:** Agreed, examples added.

**Line 72:** Agreed. We checked the reference and then changed the sentence as follows: ‘In addition to varying gear and deployment characteristics, clear differences among vessel clusters were revealed when the composition of landings was matched with the spatial distribution of longline sets.’

**Line 74:** Done

**Line 83:** Done

**Lines 85-88:** Agreed. We now start the sentence as follows: ‘Species such as blue sharks (*Prionace glauca*), shortfin makos (*Isurus oxyrinchus*) and porbeagle sharks (*Lamna nasus*) migrate freely….’

**Line 96:** Suggestion inserted.

**Line 129:** Inserted ‘observer sampling’

**Line 137:** Added ‘were’ before comprised

**Line 146:** Replaced ‘’discharged weights’ with landed weights.

**Line 148-149:** OK, done

**Line 154:** Changed ‘fish and sharks’ to ‘individuals’.

**Line 203:** Changed ‘fish and sharks’ to ‘individuals’.

**Line 206:** Noted and changed.

**Line 208:** Noted and changed.

**Line 209:** Agreed. Changed to per vessel.

**Line 231:** No change made – most vessels were sampled by the first author – therefore ‘The observer’ (singular) was retained.

**Table 2:** No change made. The range in each block (percentage of hooks set from 2013 – 2015) is already provided in the Table, and the graduated grey-scale is only used to guide the reader’s eye. We can remove the grey altogether, if needed, but prefer to keep it.

**Line 253:** Agreed. Done

**Line 257-258:** Agreed. Added the following: ‘…based on four three-monthly quarters in each year….’

**Line 265:** Lines refer to sets. Changed this as suggested.

**Line 273:** Agreed. Changed.

**Line 278:** Agreed. Done.

**Line 280:** Agreed. Done. Note that we added ‘were’ not ‘was’ because it is plural.

**Line 281:** Agreed. Done.

**Line 288:** Agreed. Scientific names added in legend.

**Line 304:** Scientific names already mentioned in lines 293-294.

**Line 305:** Noted and changed.

**Line 307:** Removed ‘respectively’.

**Line 314:** Agreed. We added ‘The ratios of retained sharks (estimated) over landed sharks (observed) (R/L ratios)….’ as a first sentence.

**Line 329:** Agreed. Both the caption and the legend of Table 3 were improved

**General:** No change made. Gear characteristics were variable between vessels and trips and changes were not always captured on data sheets. Therefore, we focussed on spatio-temporal aspects fishing effort and the relative composition of landings to explain fishing behaviour. Adding gear configuration to the study would have added another layer of complexity and uncertainty, which we doubt that the data available would have been able to support.

**Line 366:** Done. Shortfin mako now used throughout the text.

**Line 389:** No change made. Posterior analysis and ‘a posteriori’ are the same thing – in English and Latin. We prefer the simplest route here.

**Line 389-391:** See comment under General above.

**Line 402:** Done. Replaced ‘some’ with ‘an estimated’.

**Line 404:** Agreed. Added the following text and a reference for Sulikowski et al 2020: ‘The high fishing mortality estimates potentially include sharks subjectively categorized as in a ‘poor physical condition’ (i.e. assumed dead) but which survived after release (Sulikowski et al. 2020). We therefore recognize that the high fishing mortality rates shown in this study may overestimate actual mortalities from this source.’

**Lines 411:** Unclear about this. ‘In’ or ‘on’ international markets? We think it should remain ‘on’.

Differences in fishing practices among subfleets form the backbone of this study, and we have analysed spatial and temporal trends in fishing effort, the species composition of landings, discard practices at sea (i.e. which species retained and which discarded), relative CPUE and discard mortality rates by species. We are unclear as to which other fishing practices the reviewer refers to?

Gear configuration was variable, depending on sea conditions and target species, and as stated above, the data available does not support a further analysis of gear configurations / other fishing practices beyond what we have already analysed at this point. Basically, we have now identified subfleets based on when and where they fish, and what they land. A next step (outside the scope of this study) can now be to compare differences in gear and vessel configuration, and hook sizes, bait, time of setting and hauling etc. We argue that it is too much for the present study.

**Line 477:** Agreed. We edited the sentence as follows: “…because this level of shark landings is set too high to effectively limit targeting of sharks”

**Line 483:** We deleted this section (2 sentences) because it repeated the discussion in a paragraph above (lines 436 – 447 in the original manuscript). Percentages are given in that paragraph, as the reviewer requested.

**Editor:**

We have included an ethics statement and details on any permits and permissions that may have been needed for this study.

**Line by line comments**

**Line 24:** Sentence changed, please see revised manuscript with track changes.

**Lines 30-31:** Scientific names added.

**Line 35:** Noted and changed.

**Line 60:** Noted and changed.

**Line 69:** Scientific names added.

**Line 82:** Noted and changed.

**Line 85:** Noted and changed.

**Line 87:** Noted and changed.

**Line 92:** Deleted hyphen.

**Line 96:** I have removed the abbreviations. This is referring to the southwest Indian Ocean. Later on in line 122 Southwest refers to one of the geographical areas.

**Lines 101 and 102:** Removed abbreviations.

**Line 102:** Removed ‘respectively’.

**Line 107:** Changed to ‘in the National Plan of Action for South Africa’.

**Line 108:** Deleted NPOA.

**Line 119:** No this is a geographical region. Previously it was the southwest Indian Ocean.

**Line 131:** Spelled out IOTC and ICCAT

**Line 132:** Removed EEZ.

**Line 137:** Noted and changed.

**Line 197:** Removed ‘respectively’.

**Line 219:** Changed ‘recovered’ to ‘identified’.

**Line 224:** Changed ‘recovered’ to ‘identified’.

**Line 225:** Removed ‘Supplemental data’.

**Lines 234-235:** This is mentioned here because if one was to look at the raw data provided, they will see that the vessel was sampled in a different year. It is better to disclose this information here than not at all.

**Line 246:** Noted and changed.

**Line 254:** Added in ‘respectively’.

**Line 256:** Added in comma.

**Line 273:** Removed ‘respectively’.

**Line 281:** Noted and changed.

**Line 291:** Noted and changed

**Line 306:** Deleted hyphen.

**Line 311:** Deleted hyphen.

**Line 312:** Removed ‘respectively’.

**Line 336:** OK, done as follows: “For this section, sharks discarded in poor physical condition (assumed unlikely to survive) included individuals that were discarded dead (see bars on Fig 6).

**Line 346:** Bar description added to figure title.

**Line 363:** Comma deleted.

**Line 391:** Deleted hyphen.

**Line 404:** We don’t see the difference

**Line 405:** Replaced ‘caused’ with ‘incurred’.

**Line 408:** Clarified that it was both study [10] and [24].

**Line 415:** Noted and changed.

**Line 425:** Added in comma.

**Line 443:** Changed to ‘depredating’.

**Line 449:** Noted and changed.

**Line 454:** Deleted hyphen.

**Line 478:** Noted and changed.

**Line 487:** Changed ‘fishermen’ to ‘fishers’.
