# Supplementary material for: Shark discards in selective and mixed-species pelagic longline fisheries
Source: PLoS One. 2020 Aug 31;15(8):e0238595. doi: 10.1371/journal.pone.0238595 (PMC7458300; doi:10.1371/journal.pone.0238595)
Supplement: Attachment — Submitted filename: Jordaan_et_al._Response to Reviewers_Revision_2.docx [file pone.0238595.s011.docx]

**Revisions made to manuscript (PONE-D-20-12467)**

Dear Dr. Christos Maravelias,

We thank the two reviewers and yourself for many good suggestions on how to improve the manuscript. We have taken all the comments into account in the main text of the manuscript, shown by track-changes and the responses below.

As requested, we have uploaded the revised manuscript, a manuscript with track changes as well as this response to the reviewer’s comments.

We trust that you will find the revised manuscript a much-improved product, suitable for acceptance by PLoS One.

Yours sincerely,

Gareth Jordaan, Jorge Santos and Johan Groeneveld

**Reviewer 1 and Editors comments**

**Overall comments**

**Reviewer 1:** Concerns remain over the quality of the available data - only one year, not comprehensive of all quarters, not randomized, not equally distributed, etc. Improvements have been made in the manuscript by correcting errors in reported values, however, which reduced confusion regarding data exclusion. This study is of value to the field, and future work will benefit greatly from improved data collection efforts.

**Editor:** There are still concerns related to the quality of the available data. Namely, the use of only one year of data being not comprehensive of all quarters, not randomized, not equally distributed. The authors should provide further explanations on the above and make such limitations of their datasets more evident in the ms. The need for improved data collection schemes should also be highlighted.

**Authors:** We are fully aware of the quality of the data, which is unfortunately typical of data collected on commercial fishing vessels, where sampling opportunities are restricted by numerous factors. When and where a vessel goes depends on decisions taken by the fishing company, aiming to maximize catches and profits. Sailing dates are highly inconstant, depending on weather, demand for fish, vessel breakdowns or information on where fish are congregating. Vessels leave from different ports and sometimes don’t have space for an observer; at-sea decisions based on perceived fish abundance may lead to sudden changes in areas fished, or even changes in the species or sizes retained or discarded. We do not offer these as excuses – rather, they are simply the realities of fisheries-independent sampling on commercial fishing vessels. For these reasons, the data generated by fisheries-independent observers will very rarely be comprehensive of all quarters, equally distributed across fishing areas, or be completely randomised.

We did, however, use all the tools to our disposal to mitigate sampling effects. This included using a single observer on all trips (GJ, the first author of the manuscript) to collect data in a consistent way; covering as many vessels as logistically possibly to evaluate variability in discard practices; complete quarterly (seasonal) and areal (spatial) coverage of at least one subfleet (subfleet 1); undertaking a post-hoc sampling trip in 2018 to increase sample size for subfleet 2; and stressing throughout the manuscript that higher levels of observer sampling are required to increase confidence in discard ratio estimates. Indeed, in the Discussion, we even highlight the potential bias in estimates for subfleets 2-4, where sampling did not cover all quarters and areas, and recommend ways to improve estimates. We are very confident, however, in the methodology that we have developed, and were delighted to see that Reviewer 1 agrees that it will be very useful in future studies.

We have made changes to the text to highlight the limitations of the data, and stress throughout the manuscript that data collection should be improved (see highlights in attached manuscript)

**Reviewer 1:** Reviewer spent hundreds of sea days on commercial vessels, including longliners, frequently with a 90-100% sampling rate, and therefore fully appreciates (and respects) the realities of at-sea data collection.

Authors: We thank the reviewer for the clarification. In our case, the observer also undertook other tasks – such as biological sampling of the captured sharks, which prevented him from observing all the hooks that came on-board. Our sampling strategy relied on sub-sampling of sections of the line being hauled, interspersed with periods for biological sampling. Nevertheless, sampling covered 56 – 80% of hooks hauled on observed trips, which is still a very high percentage, and an acceptable subsampling coverage.

**Line by line comments (from marginal comments in pdf)**

**Line 32:** We have changed to singular, and now use the more correct term ‘fisheries-independent observer’ throughout the manuscript.

**Line 39:** Added in “among other factors”.

**Line 51:** Replaced “and” with “therefore”.

**Line 52:** Deleted “therefore”.

**Line 91:** Added comma before “which”.

**Line 145:** We have edited the text (see track changes) to clarify the processing used on the different species – (i.e. removal of head, gills, gut and fins etc.) before weighing them.

**Line 147:** The vessels that caught large numbers of shortfin makos targeted them in a known congregation area (Agulhas region) addressed in the Discussion section and also elaborated on in Groeneveld et al. 2014. Vessels actively targeting sharks (shortfin makos, in this case) have been grouped as subfleet 4, based on their landings during 2015. We have adapted the paragraph (lines 500-502 in the revised manuscript with tracked changes) to include spatio-temporal closures for shortfin mako nursery areas and not only blue shark nursery areas as stated before.

**Line 177:** See response above (for line 32)

**Table 1:** Made corrections to total catch.

**Line 235:** See response above (for line 32).

**Line 269 to 270**: Text added in the section Materials and Methods – Observer sampling to address the comments regarding addition of a sample in 2018 (See track changes).

**Line 327:** Changed “importance” to “number”.

**Line 332:** Changed “confirmed” to “supported”.

**Line 365:** This is the correct number. Correction made in lines 460 of the revised manuscript with track changes.

**Line 386:** See comment above

**Line 446 to 447:** Corrected to 69 687.

**Figure 5:** The correction was made in Table 1.
